# Replacement of Molybdenum by Tungsten in a Biomimetic Complex Leads to an Increase in Oxygen Atom Transfer Catalytic Activity

**DOI:** 10.1021/acs.inorgchem.2c01868

**Published:** 2022-07-27

**Authors:** Miljan Z. Ćorović, Fabian Wiedemaier, Ferdinand Belaj, Nadia C. Mösch-Zanetti

**Affiliations:** Institute of Chemistry, Inorganic Chemistry, University of Graz, 8010 Graz, Austria

## Abstract

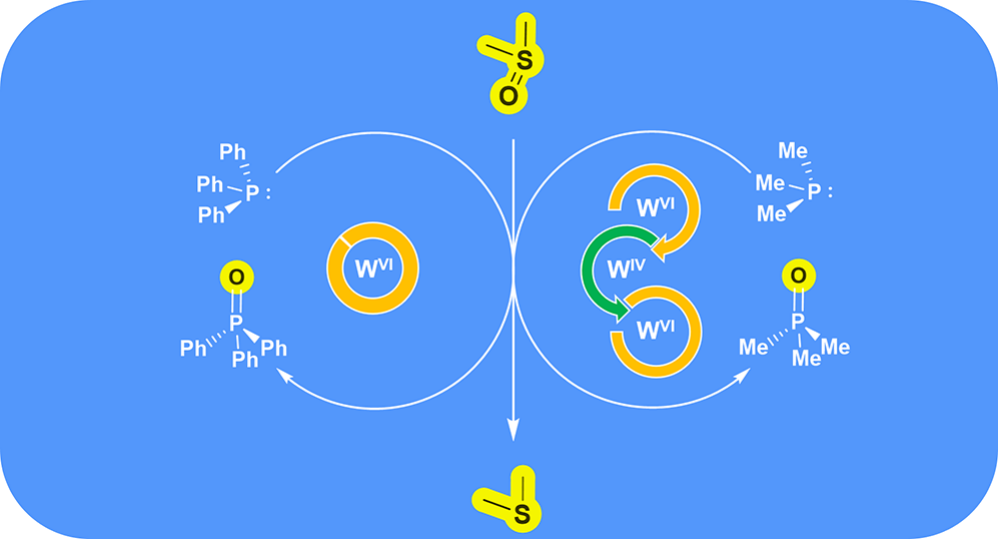

Upon replacement of molybdenum by tungsten in DMSO reductase isolated from the Rhodobacteraceae family, the derived enzyme catalyzes DMSO reduction faster. To better understand this behavior, we synthesized two tungsten(VI) dioxido complexes [W^VI^O_2_L_2_] with pyridine- (PyS) and pyrimidine-2-thiolate (PymS) ligands, isostructural to analogous molybdenum complexes we reported recently. Higher oxygen atom transfer (OAT) catalytic activity was observed with [WO_2_(PyS)_2_] compared to the Mo species, independent of whether PMe_3_ or PPh_3_ was used as the oxygen acceptor. [W^VI^O_2_L_2_] complexes undergo reduction with an excess of PMe_3_, yielding the tungsten(IV) oxido species [WOL_2_(PMe_3_)_2_], while with PPh_3_, no reactions are observed. Although OAT reactions from DMSO to phosphines are known for tungsten complexes, [WOL_2_(PMe_3_)_2_] are the first fully characterized phosphine-stabilized intermediates. By following the reaction of these reduced species with excess DMSO via UV–vis spectroscopy, we observed that tungsten compounds directly react to W^VI^O_2_ complexes while the Mo analogues first form μ-oxo Mo(V) dimers [Mo_2_O_3_L_4_]. Density functional theory calculations confirm that the oxygen atom abstraction from W^VI^O_2_ is an endergonic process contrasting the respective reaction with molybdenum. Here, we suggest that depending on the sacrificial oxygen acceptor, the tungsten complex may participate in catalysis either via a redox reaction or as an electrophile.

## Abstract 2

A W(VI) dioxido complex with pyridine-2-thiolate ligands mimics the activity of DMSO reductase. The W complex catalyzes the reactions more efficiently than the Mo analogue, similar to the catalytic behavior of DMSO reductase upon metal ion replacement. Surprisingly, the catalytic performance was higher for PPh_3_ than for PMe_3_, suggesting two different mechanisms. While with PMe_3_, reduction to a W(IV) species is observed, with PPh_3_, the W(VI) complex seems to play the role of an electrophile.

## Introduction

**Scheme 1:**
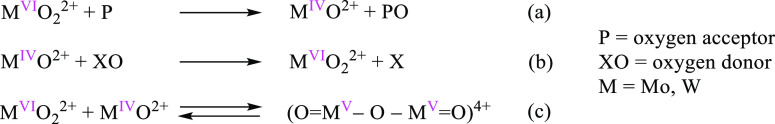
Reaction Steps in an OAT Using Model Complexes

Nature has taken advantage of minor differences in the coordination chemistry of molybdenum and tungsten to perform several sophisticated enzymatic transformations. The physicochemical properties of their bioavailable (oxo)anions, MO_4_^2–^ (M = Mo, W), allow similar odds for both metals to incorporate into the enzyme structure. Generally, in tungsto- and molybdoenzymes, the metal center in oxidation state +IV or +VI is coordinated by one or two of any variations of the metallopterin moiety.^1,2^ However, the activity of the metalloenzymes is strongly dependent on the metal ion situated in their active site.^3,4^ Some molybdoenzymes, such as sulfite oxidase^5^ or nitrate reductase,^6−8^ are less active or not active at all upon replacement of Mo by W. Conversely, when molybdenum is replaced with tungsten in DMSO reductase (DMSOr) from *Rhodobacter capsulatus* or *Rhodobacter sphaeroides*, the derived enzyme reduces DMSO at a higher rate but also is inactive in catalyzing the reverse DMS oxidation.^9,10^ Similarly, trimethylamine−*N*–oxide reductase (TMAOr) from *Escherichia coli* shows a slight increase in catalytic activity when molybdenum is substituted by tungsten.^11^ Both DMSOr and TMAOr catalyze biochemical transformations known as oxygen atom transfer (OAT) which are widespread reactions for molybdenum and tungsten oxidoreductase enzymes.^12,13^ To better understand the mechanism under which the metalloenzymes perform the OAT, many molybdenum model compounds were synthesized and investigated, while tungsten modeling chemistry is far less explored.^1,14,15^ Frequently used model reactions for OAT usually employ the biological substrate DMSO and different tertiary phosphines as sacrificial oxygen acceptors.^16−18^ In general, if the catalyst is based on a higher-valent metal center (M^VI^O_2_^2+^), OAT model reactions involve the concomitant two-electron reduction coupled with oxygen abstraction by the oxygen acceptor (Scheme 1a).^16^ Reduced species (M^IV^O^2+^) can further undergo oxidation to recover the catalyst by abstracting the oxygen from DMSO or any related substrate (Scheme 1b).^19^

Enzyme-like reactivity was achieved with different model catalysts, most frequently dithiolene-based molybdenum complexes due to their resemblance to the biological active sites.^20−22^ However, there are still many limitations to overcome since dithiolene-based ligands often cause the formation of tris chelate compounds with molybdenum and tungsten in oxidation states +IV or +V.^1^ Another issue in OAT modeling chemistry with molybdenum is the formation of relatively inert Mo(V) μ-oxo dimers, which are formed upon comproportionation of a Mo(IV) and Mo(VI) species (Scheme 1c).^4,14^ These dimers may still support OAT reactions but often decrease the catalytic performance depending on the position of the equilibrium. While special care must be taken for the analysis of the equilibrium,^23^ it may be regulated by the ligands’ steric and electronic properties.^24,25^ Thus, the dinuclear Mo(V) species may be viewed as an “electronic buffer,” where the two electrons are each localized on a single molybdenum center.^23^

Tungsten(VI) models in OAT reactions are studied less and mainly alongside analogous molybdenum compounds. Such investigations revealed that analogous Mo(IV, VI) and W(IV, VI) complexes are nearly isostructural;^4^ however, lower or no catalytic activity of the tungsten system is usually observed.^26−28^ This may mainly be explained by the half-reaction of the catalytic cycle requiring reduction of the metal center (Scheme 1a), which is often challenging with tungsten due to its lower redox potential.^29−31^ Possibly for these reasons, W(V) μ-oxo dimers are only known with scorpionate^32,33^ and dithiocarbamate ligands,^34,35^ besides an organometallic example.^36^ Comparative studies revealing a higher OAT activity of the tungsten(VI) compound than that of the molybdenum analogue are extremely rare. Some years ago, we described Mo(VI) and W(VI) complexes with a [ONN] donor, which catalyzed the OAT from DMSO to PMe_3_, and found the higher homolog to be significantly more efficient.^37^ However, reduction of the tungsten(VI) center was not observed even with an excess of phosphine, which made us consider retention of the oxidation state throughout the catalytic cycle. For this reason, additional studies should be carried out with tungsten(VI) compounds. Furthermore, they are generally more stable at elevated temperatures and show lower affinity toward μ-oxo dimers formation.

We recently described functional DMSO reductase models of the type [MoO_2_L_2_] with bidentate monoanionic pyridine/pyrimidine-2-thiolate ligands. Those complexes react with PMe_3_ and PPh_3_, yielding Mo(IV) and Mo(V) species, respectively.^38^ Here, we present the challenges and results of replacing the molybdenum with tungsten in analogous complexes.

## Results and Discussion

### Synthesis and Characterization of Tungsten (VI) Dioxido Complexes

**Scheme 2:**
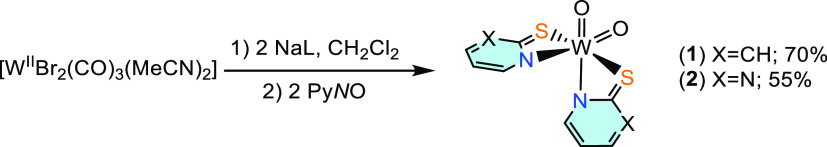
Two-Step Synthesis of [WO_2_L_2_] Complexes

Two tungsten(VI) dioxido complexes [WO_2_L_2_] (L = pyridine-2-thiolate (PyS), (**1**) and pyrimidine-2-thiolate (PymS), (**2**)) were synthesized in two steps starting from the tungsten(II) precursor [WBr_2_(CO)_3_(MeCN)_2_] (Scheme 2). After reacting the precursor with 2.05 equiv of the ligand salt NaL in CH_2_Cl_2_ to obtain related tricarbonyl complexes [W(CO)_3_L_2_], the reaction mixture was filtered and reacted with two equiv of pyridine-*N*-oxide overnight. After concentrating the solution and adding MeCN, dark yellow microcrystals of the products were collected in good yields directly from the reaction flask upon cooling or slow solvent removal.

Alternatively, synthesis of [WO_2_L_2_] could also be performed starting from tungsten(VI) precursors [WO_2_Cl_2_(dme)] (dme = dimethoxyethane) and [WO_2_(acac)_2_] (acac = acetylacetonate) at −10 °C via salt metathesis or ligand substitution, but many impurities complicate the work-up. For the synthesis of **2**, inert conditions are crucial because otherwise almost immediate ligand hydrolysis occurred accompanied by the formation of the tungsten(IV) species [W(PymS)_4_] (together with the disulfide of the ligand).^39^

**Figure 1:**
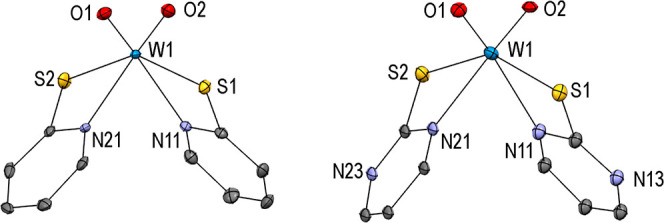
Molecular structures (50% probability thermal ellipsoids) of complexes **1** (left) and **2** (right) showing the atomic numbering scheme. H atoms are omitted for clarity.

Complex **1** is soluble, while complex **2** has low solubility in chlorinated solvents. Low solubility in MeCN and hydrocarbons was observed for both complexes. Although stable in solid-state for a few days under ambient conditions, solutions of **1** and **2** are sensitive to moisture, and syntheses were successful only under strictly inert conditions. The dioxido complexes were nevertheless isolated in pure form and fully characterized. ^1^H NMR and ^13^C NMR spectroscopy revealed the existence of only one isomer in solutions of **1** and **2** and, together with elemental analysis, confirmed the purity of the samples. IR signals deriving from W=O bonds were detected in a range of 902–953 cm^–1^, similar to other neutral W^VI^O_2_ complexes.^40−42^ Compounds **1** and **2** were crystallized from CH_2_Cl_2_/MeCN at −37 °C to obtain a single crystal suitable for X-ray diffraction analysis. Experimental details and structure refinements are reported within the Supporting Information. Molecular views are presented in Figure 1.

Both W^VI^O_2_ complexes crystallized as single isomers with sulfur atoms oriented *trans* and nitrogen atoms *cis* to each other and *trans* to oxygen atoms. The structures are isotypic with the published molybdenum analogues,^38,43,44^ with metal–oxygen double bonds slightly elongated for the tungsten analogues (W–O bonds range: 1.715–1.743 Å; Mo–O bonds range: 1.693–1.711 Å), as previously observed in the literature.^45^ This is certainly due to the differences in the radial distribution functions of the Mo and W orbitals involved in the bonding. Single crystals of compound **2** reveal two conformers, where the angle between the two least–square planes of the two ligands varies [71.1(5) and 75.4(5)°].

### W versus Mo in OAT Catalysis

To test the catalytic activity of complexes **1** and **2**, identical experimental conditions were used as in our previous molybdenum OAT work allowing direct comparison.^38^ Accordingly, well-dried deuterated DMSO was used as an oxygen donor and solvent, while PMe_3_ or PPh_3_ were used as oxygen acceptors. To remove all solvent residues, complexes were dried in vacuo at 50 °C for at least 5 h before use. Catalyst loading was 1 mol % calculated versus phosphines used as limiting reagents. The formation of the respective phosphine oxides was followed via ^1^H and ^31^P NMR spectroscopy at rt, and all the samples were prepared in J. Young NMR tubes to provide a water- and air-free environment. Blank experiments revealed that complex **2** is not stable in the DMSO-*d*_6_ solution because of decomposition to the disulfide (PymS)_2_ (^1^H NMR (300 MHz, DMSO-*d*_6_): 8.71 (d, 4H), 7.37 (t, 3H)), DMS-*d*_6_ and presumably WO_3_. The formation of (PymS)_2_ was confirmed by independent synthesis as described in the Supporting Information.^46^ Furthermore, a ^1^H NMR spectrum of a CDCl_3_ solution of **2** with an excess of DMSO revealed signals in the region 1.85–2.07 ppm for DMS. Nevertheless, a comparison between tungsten and molybdenum catalysts was possible for the pyridine-2-thiolate system [MO_2_(PyS)_2_] (M = Mo or W), as shown in Table 1.

**Table 1:** Results of Catalytic OAT Reactions between DMSO and PPh_3_ and PMe_3_^a^

catalytic loading and catalyst	conversion (%)	time
PMe_3_ → OPMe_3_		
1 mol % [MoO_2_(PyS)_2_]	100	>2 weeks
1 mol % [WO_2_(PyS)_2_]	100	5 h
PPh_3_ → OPPh_3_		
1 mol % [MoO_2_(PyS)_2_]	100	48 h
1 mol % [WO_2_(PyS)_2_]	100	3.5 h

a Conditions: DMSO-*d*_6_ (0.5 mL), PPh_3_ (114 μmol) or PMe_3_ (233 μmol), and catalyst (1 mol % vs PPh_3_ or PMe_3_). Full conversion of PPh_3_ to OPPh_3_ and PMe_3_ to OPMe_3_, respectively, was determined by NMR spectroscopy. All experiments were performed at least three times. In blank experiments without a metal complex, no conversion of phosphines was observed.

As summarized in Table 1, OAT experiments reveal significantly higher activity of the tungsten compound, both with PMe_3_ and PPh_3,_ respectively, compared to molybdenum. Surprisingly, the more basic PMe_3_ was less efficiently converted than PPh_3_, suggesting different mechanisms between the two substrates. The higher activity of the tungsten compound is unexpected since, in most comparative literature studies, tungsten complexes were either less efficient or not active at all.^17,26,27,41,47^ Indeed, if considering a typical OAT mechanism,^13^ which includes the reduction of a metal center M(VI) to M(IV), molybdenum catalysts are expected to be faster due to their favorable redox potentials.^31^ In the tungsten-catalyzed experiment with PMe_3_, the yellow color of the DMSO solution of **1** changed initially to dark green, indicating that the mechanism occurs via reduction, identical to the suggested molybdenum-based pathway. On the other hand, no color change was observed during the tungsten-catalyzed experiments with PPh_3_. To further elucidate this unusual behavior in catalysis, the reactivity of complexes **1** and **2** toward phosphines was investigated in absence of DMSO.

### Reactivity of [WO_2_L_2_] toward Phosphines

**Scheme 3:**
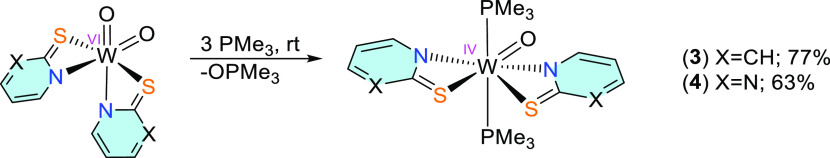
Reduction of [WO_2_L_2_] with PMe_3_ in Chlorinated Solvents at rt

Although [MoO_2_L_2_] complexes react with PPh_3_ yielding μ-oxo dimers [Mo_2_O_3_L_4_],^38^ tungsten complexes **1** and **2** do not react with PPh_3_ under the same conditions. Also, with longer reaction times (24 h) and the use of various solvents (CD_2_Cl_2_, MeCN-*d*_3_, and C_6_D_6_), no OPPh_3_ was observed, as evidenced by ^1^H and ^31^P NMR spectroscopy. On the other hand, both complexes react with 3 equiv of the more electron-rich phosphine PMe_3_ overnight (Scheme 3) under the formation of the seven-coordinated reduction products [WO(PMe_3_)_2_L_2_] (L = PyS **3**, PymS **4**) and OPMe_3_. They can be isolated in good yields after work-up as described in the Supporting Information as dark green (**3**) or violet (**4**) microcrystals. Both compounds contain two PMe_3_ ligands in *trans* position to each other. Such a stabilization, which is not possible with PPh_3_ due to steric hindrance, allows the reduction of the metal center with the sterically less demanding PMe_3_. Tungsten(IV) oxido compounds with two *trans* phosphine ligands that are obtained via OAT from tungsten(VI) dioxido complexes have as yet not been described.

However, two *trans*-oriented phosphines stabilizing a d^2^ W center were observed in [WO(PMe_2_Ph)_2_L] (L = 2,2′:6′,2″:6″,2‴-quaterpyridine)^48^ and [W(O)Cl_2_(CO)(PMePh_2_)_2_],^49^ but they were not studied in the context of OAT. The monophosphine complex [WO(S_2_CN(CH_2_Ph)_2_)_2_(PMe_3_)] was also described, but structural data is lacking.^35^ None of the mentioned examples were prepared by synthetic routes, including the reduction of a tungsten(VI) species.

Compounds **3** and **4** are very well soluble in chlorinated hydrocarbons, MeCN, and THF and poorly soluble in hydrocarbons and diethyl ether. The complexes are stable in chloroform for several days, unlike the molybdenum variants.

**Figure 2:**
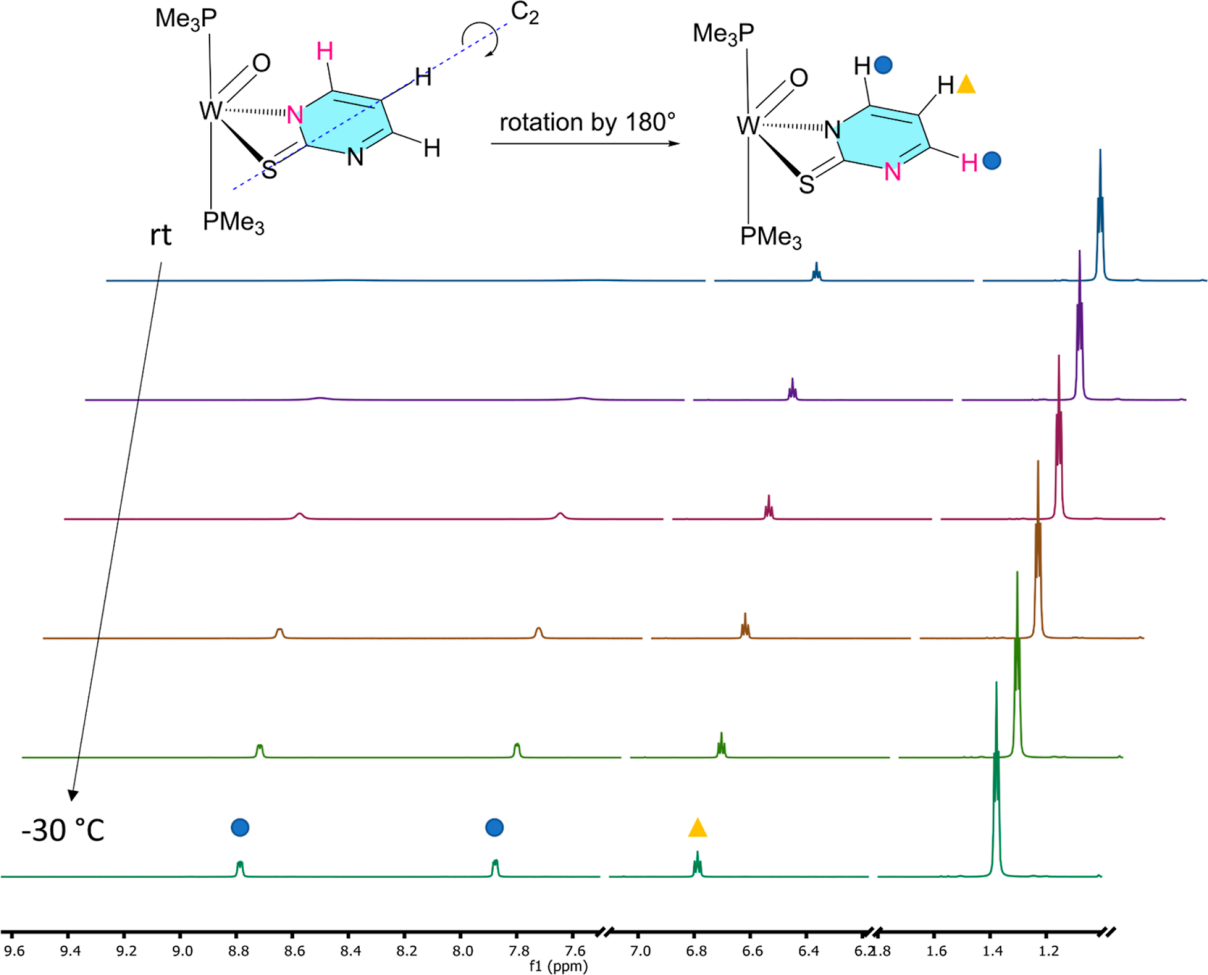
VT ^1^H NMR spectra of **4** in CDCl_3_ (measured at rt, 10, 0, −10, −20, −30 °C). The scheme represents the rotation about the C_2_ axis within the coordinated ligand. The second ligand was omitted for better visualization.

IR spectroscopy revealed a strong band at 940 cm^–1^ for both **3** and **4**, indicating the existence of a W≡O bond, which is following molybdenum analogues^38^ and related W(IV) species.^50^ To obtain meaningful NMR data for complex **4**, it was necessary to perform variable-temperature NMR experiments due to dynamic behavior (Figure 2). At room temperature, two broad signals and one sharp triplet appear in the aromatic region. At −30 °C, free rotation of the coordinated pyrimidine-2-thiolate ligand about its C_2_ axis is hindered, revealing sharp signals for all aromatic protons. The rotation is possible since the W–N bond is rather weak, which is consistent with the observed lower stability of the pyrimidine system in DMSO. Furthermore, decoordination is feasible in the presence of a π–donor oxido ligand.

^1^H NMR spectra show that **3–4** exist as single isomers in solution. Owing to virtual coupling, protons belonging to two *trans*-coordinated PMe_3_ appear as virtual triplets resonating at ≈1.36 ppm and integrating for 18H. Also, carbons belonging to coordinated PMe_3_ show triplets in the ^13^C NMR spectra. [MOL_2_(PMe_3_)_2_] (M = Mo, W; L = PyS, PymS) are isotypic for both metals allowing comparison of NMR data (Table 2).

**Table 2:** Chemical Shifts (ppm) of H and P Atoms in the Analogous W^IV^ and Mo^IV^ Complexes^a^

δ (ppm)^a^	^1^H (PyS/PymS)	^1^H (PMe_3_)	^31^P{^1^H}	refs
[WO(PyS)_2_(PMe_3_)_2_] (3)	8.68, 6.87, 6.69, 6.49	1.33	–27.51	
[MoO(PyS)_2_(PMe_3_)_2_]	8.73, 7.16, 6.63, 6.56	1.24	–8.07	(38)
[WO(PymS)_2_(PMe_3_)_2_] (4)	8.73, 7.89, 6.74	1.34	–27.66	
[MoO(PymS)_2_(PMe_3_)_2_]	8.56 (4H), 6.66	1.25	–9.00	(38)

a NMR spectra were recorded in CD_2_Cl_2_ and at rt.

The effect of metal ion replacement is observable upon a comparison of the ^31^P{^1^H} NMR spectra: signals belonging to PMe_3_ coordinated to Mo are downfield shifted by approx. 20 ppm due to the lower π basicity of the metal. Furthermore, signals of the tungsten compounds **3** and **4** reveal ^183^W satellites, which are absent in the Mo complexes.^38^

**Figure 3:**
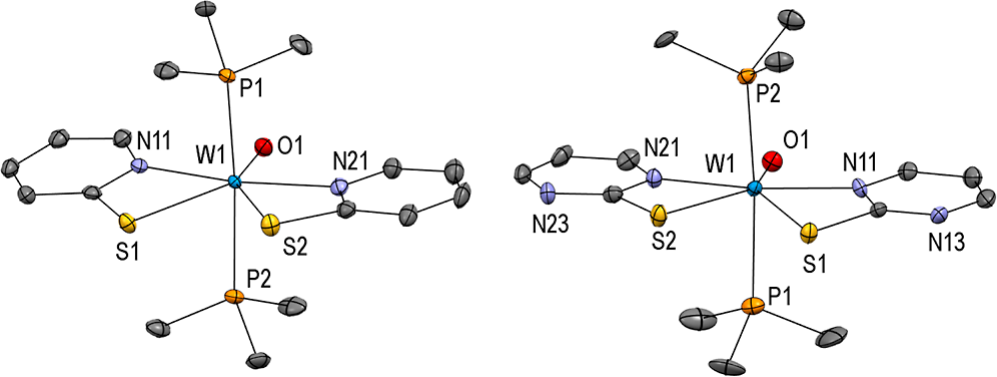
Molecular structures (50% probability thermal ellipsoids) of complexes **3** (left) and **4** (right) showing the atomic numbering scheme. H atoms are omitted for clarity.

Compound **3** crystallized from a CH_2_Cl_2_/*n*-heptane mixture at −37 °C, while compound **4** crystallized from a saturated MeCN solution, forming single crystals suitable for X-ray diffraction analysis. Molecular views are presented in Figure 3, and selected bond lengths and angles are shown in Table 3.

**Table 3:** Selected Bond Lengths (Å) and Angles (°) of [M^IV^OL_2_(PMe_3_)_2_] (M = W, Mo; L = PyS (**3**); PymS (**4**))

	**3**	**4a**	[MoO(PyS)_2_(PMe_3_)_2_]^38^	[MoO(PymS)_2_(PMe_3_)_2_]^38^
M–O	1.745(2)	1.727(3)	1.7190(11)	1.7115(13)
M–S1	2.6675(8)	2.6825(13)	2.6906(4)	2.6880(5)
M–S2	2.6668(10)	2.6399(12)	2.6891(4)	2.7006(5)
M–N11	2.205(3)	2.162(4)	2.2228(13)	2.2084(17)
M–N21	2.195(3)	2.181(4)	2.2234(13)	2.2072(16)
M–P1	2.4760(8)	2.4909(14)	2.4910(4)	2.5023(5)
M–P2	2.4883(9)	2.4936(14)	2.4994(4)	2.5080(6)
P1–M1–P2	170.08(3)	170.96(4)	168.946(14)	169.654(19)

The crystal structure analysis of **3** and **4** revealed a pentagonal bipyramidal surrounding of the W atom with two PMe_3_ ligands *trans* to each other, confirming ^1^H NMR data. The asymmetric unit of **4** consists of two complexes (**4a** and **4b**) with the same connectivity and slightly different geometrical parameters. Here, only data of **4a** are discussed, while those of **4b** are given in the Supporting Information (Table S6).

Compounds **3** and **4** are isostructural to previously described molybdenum versions.^38^ As expected, the M=O bonds are slightly longer in the higher homolog. Moreover, all listed complexes have rather large M–S distances compared to previously described complexes with pyridine and pyrimidine-2-thiolate ligands,^51,52^ which is presumably due to the higher coordination number. However, the metal–sulfur distances are shorter than in reported Mo/W complexes with thioether ligands.^42,45,53^

### Mechanistic Insights into OAT Catalysis with PMe_3_

The proposed cycle for [MoO_2_L_2_]-catalyzed OAT suggests three steps: (1) reduction of the starting compound with 3 equiv of PMe_3_ to stable 18e^–^ species [MoOL_2_(PMe_3_)_2_], (2) reversible dissociation of two *trans*-coordinated PMe_3_ to form catalytically active [MoOL_2_], and (3) reoxidation to the starting [MoO_2_L_2_] with DMSO and formation of DMS.^38^ The first step is favorable for Mo compounds since the redox potential of Mo(VI)/Mo(IV) is usually higher than for W(VI)/W(IV).^29^ Moreover, the time necessary to reduce M^VI^O_2_ to [MOL_2_(PMe_3_)_2_] (M = Mo or W) with PMe_3_ is shorter for the Mo variants (3 h for Mo vs 16 h for W), evidenced by comparing synthetic procedures (see the Supporting Information and previous publication^38^). Here, we followed the oxidation step via UV–vis spectroscopy. Excess DMSO (1000 equiv) was added to a CH_2_Cl_2_ solution of the respective [MO(PyS)_2_(PMe_3_)_2_] complex at room temperature, and data were acquired until complete conversion (Table 4).

**Table 4:** Reaction Time for the Oxidation of [MO(PyS)_2_(PMe_3_)_2_] with 1000 equiv of DMSO to [MO_2_(PyS)_2_]^a^

compound	reaction completed after (h)
[MoO(PyS)_2_(PMe_3_)_2_]	9
[WO(PyS)_2_(PMe_3_)_2_] (**3**)	5

a Conditions: to a 3 mL of CH_2_Cl_2_ solution of the [MO(PMe_3_)_2_(PyS)_2_] (0.3 μmol) in a quartz cuvette, 1000 equiv of DMSO was added in the glove box. UV–vis measurement started 3 min after the preparation of the sample. The screening was performed at 25 °C.

**Figure 4:**
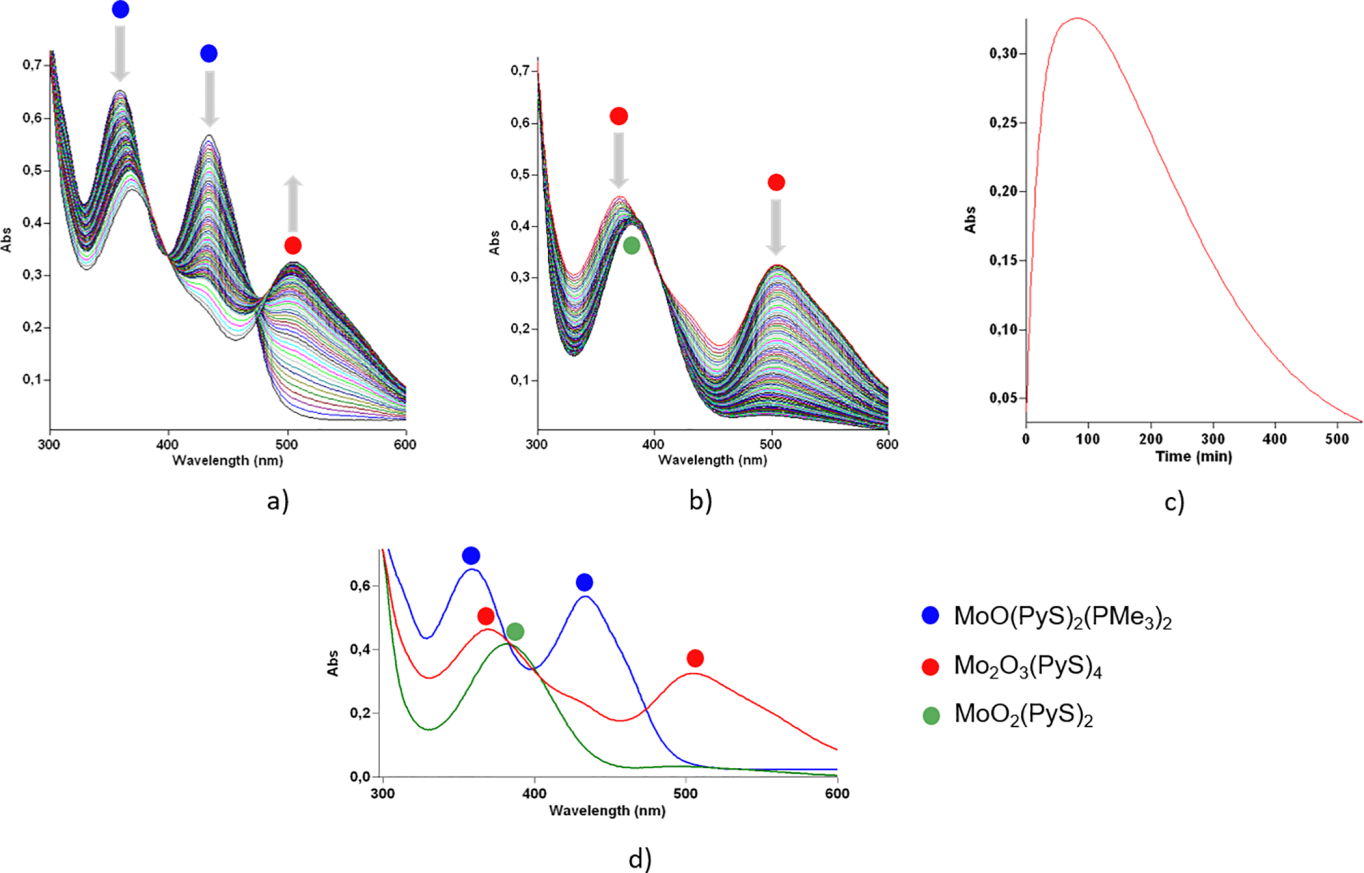
(a) UV–vis spectra of the reaction of [MoO(PyS)_2_(PMe_3_)_2_] (λ_1_max = 360 nm; λ_2_max = 435 nm) to [Mo_2_O_3_(PyS)_4_] (λ_1_max = 370 nm; λ_2_max = 505 nm) during the first 1.5 h; (b) UV–vis spectra of the reaction of [Mo_2_O_3_(PyS)_4_] with DMSO forming [MoO_2_(PyS)_2_] (λmax = 385 nm) during the following 7.5 h; (c) absorbance at 505 nm over 9 h; (d) UV–vis spectra at the beginning (blue), after 1.5 h (red), and after 9 h (green) of the reaction.

As expected, reoxidation of W(IV) to W(VI) with DMSO is faster by a factor of 1.8 compared to Mo, which is similar to the oxidation rate differences observed for dithiolene-based M^IV^O complexes.^54^ However, a detailed analysis of the UV–vis spectra reveals significant differences between the two metals (Figure 4). Namely, the Mo^IV^O complex does not simply react to the respective Mo^VI^O_2,_ but an intermediate species is formed after 1.5 h of reaction (Figure 4a). This species with λ_max_ values at 370 and 505 nm we found to be the dinuclear molybdenum(V) compound [Mo_2_O_3_(PyS)_4_], previously reported as a product of the reduction of Mo^VI^O_2_ with PPh_3_.^38^ Such dimers are common in molybdenum oxido chemistry.^55−57^ Here, it further reacts slowly with an excess of DMSO, yielding the oxidized Mo^VI^O_2_ complex (Figure 4b). [Mo_2_O_3_(PyS)_4_] is poorly soluble in any solvent, precluding NMR spectroscopic observation. To confirm that the dimer is formed before the conversion to the dioxido complex, [MoO(PyS)_2_(PMe_3_)_2_] was reacted with 1 or 2 equiv of DMSO in CD_2_Cl_2_ in a J. Young tube, which caused crystallization of [Mo_2_O_3_(PyS)_4_] in pure form in both cases.^1^H NMR spectra of the solution revealed the formation of DMS and OPMe_3_, while no traces of the dioxido complex were observed. Dimer formation gives evidence that the short-living species [MoOL_2_] is indeed formed in the solution, which immediately reacts with [MoO_2_L_2_]. Thus, the dimer may be considered a resting state of the catalytic cycle. The oxidation rate of the molybdenum dimer is quite low (Figure 4b,c), which is influenced by the dissociation barrier^38^ and the low solubility.

In contrast, the formation of a dimer was not observed by UV–vis spectroscopy when reacting tungsten compound **3** with an excess of DMSO (Figure S17), but rather the oxidized tungsten(VI) complex [WO_2_(PyS)_2_] is formed directly. In the reaction of **3** with DMSO, one PMe_3_ may dissociate first, leaving a vacant site for DMSO to interact with the complex and transfer an oxygen atom, causing the second PMe_3_ to leave. Thus, phosphine-free [WOL_2_] is presumably never formed, so W dimer formation is not observed in contrast to molybdenum. Additionally, tungsten dimer formation might not be possible here because W^VI^O_2_ is not a good enough oxidant for W^IV^O due to unfavorable differences in redox potentials of the W^VI^/W^V^ and W^V^/W^IV^ couples. However, the flexibility of the [MOL_2_] core with bidentate ligands (L = PyS, PymS) allows the isolation of the reduced product. In this case, the angle between the two least–square planes of the ligands in [MO_2_L_2_] expands from 71.1(5)° (L = PymS) or 80.33(15)° (L = PyS) to an almost coplanar geometry in [MOL_2_(PMe_3_)_2_]. We assume that the stabilization by two *trans* PMe_3_ molecules is crucial for the isolation of reduced species and that, therefore, a lack of flexibility in other ligand systems prevents the formation of phosphine-stabilized OAT intermediates for tungsten. Such a rearrangement seems unlikely in the enzymes where the two metalloprotein ligands are locked into a pseudo-*cis* orientation by a plethora of hydrogen bonds.^58−61^

### Suggested OAT Mechanism with PPh_3_

**Scheme 4:**
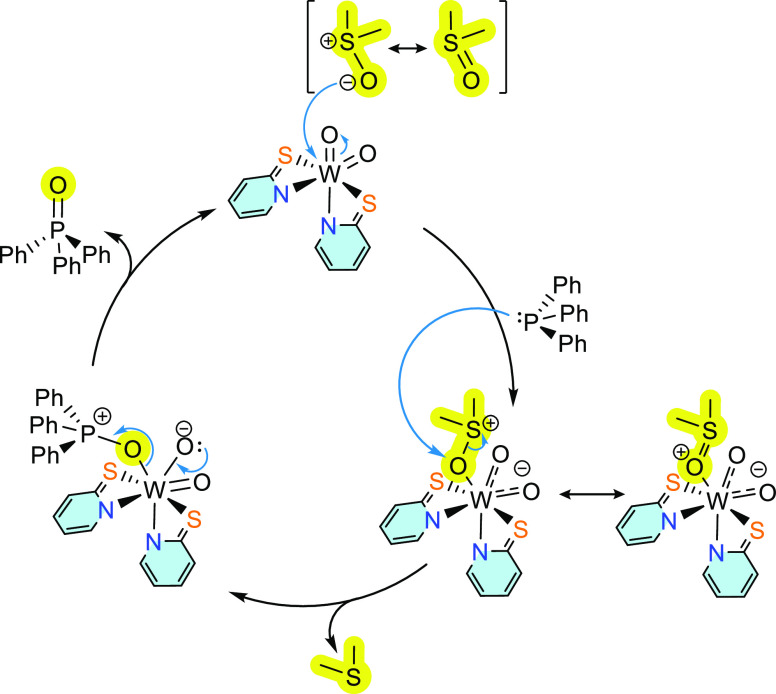
Proposed OAT Mechanism Catalyzed by Complex **1**

As described above, dioxido compounds **1** and **2** do not react with PPh_3_ while nevertheless catalyzing OAT with DMSO. For this reason, an alternative mechanism with PPh_3_ versus PMe_3_ seems likely. Since no reduction of the tungsten center was observed during catalysis, the mechanism presented in Scheme 4 is suggested. Accordingly, the tungsten center enables polarization of the DMSO molecule under the formation of a 7-coordinate intermediate stabilized by delocalization of the charge over two W–O double bonds. The electrophilic metal center renders the oxygen atom at DMSO prone to a nucleophilic attack by phosphine. Upon elimination of phosphine oxide, the catalyst is recovered. The suggested cycle avoids the reduction of the third-row metal tungsten. We have previously observed such behavior where tungsten(VI) dioxido compounds were not reduced by PMe_3_ but still catalyzed OAT.^37^

With the second-row metal molybdenum reduction with PPh_3_ is possible, so the mechanism via the μ-oxo dimer analogous to the one with PMe_3_ is favored.

A mechanism for the W-catalyzed PPh_3_ oxidation with DMSO via the reduction of the tungsten center could, in principle, be envisioned via a transient [WOL_2_] species which is too short-lived to detect and also to form the dimer. However, our NMR experiments (*vide supra*) revealed no traces of reduction in the absence of an oxygen donor, and our calculations (*vide infra*) reveal high energy for obtaining [WOL_2_], arguing against such a mechanism.

The suggestion that OAT with W enzymes might not proceed via reduction and oxidation of the metal is intriguing and should be compared with the discussed mechanism of tungsten-dependent acetylene hydratase (AH).^2^ The latter, exhibiting a similar active site as the OAT enzymes, catalyzes the hydration of acetylene, a nonredox reaction. The tungsten center is proposed to remain in the oxidation state +IV throughout the catalytic cycle. This suggests that W may act as an electrophile, both in AH and certain OAT enzymes. Owing to size restrictions in the active site of DMSO reductase, the substrate could be polarized and further reduced without formation of an intermediate 7-coordinate species.

### Theoretical Calculations

**Scheme 5:**
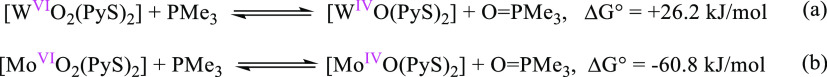
Gibbs Free Energies for the Abstraction of One Oxygen Atom from the Respective Dioxido Complexes by PMe_3_

To better understand the general reluctance of tungsten oxido complexes to form the μ-oxo dimeric species, Δ*G*° values for the abstraction of one oxygen atom by a phosphine molecule were determined by density functional theory (DFT) calculations. To simplify the computation, PMe_3_ was considered as an oxygen acceptor instead of the larger PPh_3_. Calculated Gibbs free energies are given in Scheme 5.

**Scheme 6:**
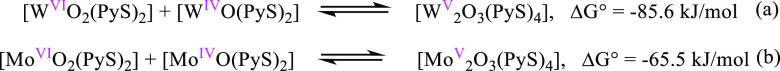
Gibbs Free Energies for the Formation of Virtual W^V^ and the Isolated Mo^V^ Dimer

Δ*G*° values reveal that the oxygen atom abstraction from the tungsten compound is an endergonic process. In contrast, the negative value in the case of molybdenum indirectly supports the fact that dimers are formed effortlessly. Results reveal that the W^IV^O complex is a high-energy species and that even the easy formation of O=PMe_3_ does not deliver enough energy to compensate. However, the coordination of two molecules of PMe_3_ seems to stabilize the coordination core, as we were able to isolate compounds of the type [WOL_2_(PMe_3_)_2_]. We also calculated the Δ*G*° values for the formation of dimers from corresponding M^IV^O and M^VI^O_2_ compounds (Scheme 6). While the molybdenum dimer [Mo_2_O_3_(PyS)_4_] is an isolated species, calculations with the analogous tungsten dimer [W_2_O_3_(PyS)_4_] are virtual.

These results reveal that if [WO(PyS)_2_] exists in solution, dimerization should occur easily, suggesting that the phosphine-free W(IV) species is never formed. This also supports the suggestion that during the oxidation of [WOL_2_(PMe_3_)_2_], first, one phosphine is detached, and the second one dissociates only after the monophosphine species [WOL_2_(PMe_3_)] interacts with DMSO. We assume that the [WOL_2_(PMe_3_)] is not a powerful enough reductant to reduce the dioxido complex and form the dimer. Moreover, the stability of [WO_2_(PyS)_2_] might be additionally enhanced by the sulfur ligands. Indeed, the average M–S bonds are shorter for the monomers than for the dimers.^38,62,63^ Other comparative studies with Mo and W compounds reach similar conclusions. For example, [MO_2_Cl_2_(mbipy)] (M = Mo, W; mbipy: 5,5′-dimethyl-2,2′-dipyridyl) reacts with 2 equiv of thiophenol in basic conditions to form [WO_2_(SPh)_2_(mbipy)] or [Mo_2_O_2_(μ-O)_2_(SPh)_2_(mbipy)_2_], wherein the latter case reduction to Mo^V^ is observed under formation of disulfide.^62^ Since dimerization, in this case, includes M–S bond formation and breaking, the authors suggest that the tungsten compound resists reduction and dimerization due to the high stability of W–S bonds.

All this suggests that the higher activity of the tungsten catalyst derives from the reluctance of dimerization or, in other words, the lower activity of molybdenum derives from the ease of dimerization, hindering further reactivity.

## Conclusions

Biomimetic tungsten(VI) dioxido complexes with the pyridine-2-thiolate ligand (PyS) and the pyrimidine-2-thiolate ligand (PymS) were synthesized and fully characterized. The OAT catalytic experiments revealed that [WO_2_(PyS)_2_] catalyzes the OAT from DMSO to PMe_3_ or PPh_3_ faster than the analogous molybdenum compound. In similar studies with [WO_2_(PymS)_2_], decomposition is observed upon dissolving in DMSO under the formation of the disulfide (PymS)_2_. Both the Mo and W complex reacted with PMe_3_, yielding a rare pentagonal bipyramidal M^IV^O species stabilized by two PMe_3_ molecules. Such a reduction requires expansion of the angle enclosed by the two least–square planes of the two aromatic ligands, which is easily occurring with the here used flexible ligands. These phosphine-stabilized species are models for the reduced form of the DMSO reductase active site and have as yet not been observed as intermediates in the oxygen transfer catalytic cycles for tungsten. When comparing the behavior of W^IV^O and Mo^IV^O species in the presence of DMSO via UV–vis spectroscopy, we observed that the W^IV^O species is directly oxidized to the corresponding W^VI^O_2_ compound, while the Mo^IV^O species first yields [Mo_2_O_3_(PyS)_4_], which further reacts to the related Mo^VI^O_2_ complex. The higher tendency of Mo compounds to form μ-oxo dimers and their low solubility decelerate the OAT catalysis in this case. This is supported by DFT calculations, which confirmed that, unlike the tungsten variant, the oxygen transfer from [MoO_2_(PyS)_2_] to PMe_3_ is an exergonic process. In the case of OAT catalysis with PPh_3_, the molybdenum variants are reduced to the respective μ-oxo dimers, while no reduction was observed for any of the tungsten complexes. However, catalytic studies showed a higher performance of the tungsten compound, presumably due to the mechanism under the retention of the metal’s oxidation state +VI. This is biologically relevant as it delivers a possible explanation for the higher activity upon replacement of Mo by W in certain enzymes: Depending on both the substrate and the ligand set, the metal may participate in catalysis either through a net redox reaction (e.g., DMSO reductase) or by serving as an electrophile for the substrate (e.g., AH). In AH, retention of the oxidation state of tungsten is proposed, suggesting that the metal may act as an electrophile under certain circumstances rather than a redox center. Furthermore, this unexpected catalytic behavior and the fact that tungsten compounds are more temperature tolerant renders the far less explored metal in atom transfer catalysis a promising candidate for future investigation.

## Supporting Information Available

Complete X-ray data, NMR spectra, UV data, and experimental procedures (PDF)
