# Supplementary material for: Replacement of Molybdenum by Tungsten in a Biomimetic Complex Leads to an Increase in Oxygen Atom Transfer Catalytic Activity
Source: Inorg Chem. 2022 Jul 27;61(31):12415–24. doi: 10.1021/acs.inorgchem.2c01868 (PMC9367641; doi:10.1021/acs.inorgchem.2c01868)
Supplement: Supplementary file 1 — ic2c01868_si_001.pdf [file ic2c01868_si_001.pdf]

Supporting Information

Replacement of Molybdenum by Tungsten in a Biomimetic Complex Leads to an Increase in Oxygen Atom Transfer Catalytic Activity

Miljan Z. Ćorović,[a] Fabian Wiedemaier,[a] Ferdinand Belaj,[a] Nadia C. Mösch-Zanetti[, a]*

[a]*Institute of Chemistry, Inorganic Chemistry, University of Graz, 8010 Graz, Austria*

* Corresponding author. Tel.: +43 (0)316 380 – 5286

E-mail address: nadia.moesch@uni-graz.at

Table of Contents

1 General Considerations S1
2 DFT calculations S2
3 Syntheses S3
4 Crystal Structure Determination S5
5 NMR spectra S14
6 References S25

1 General Considerations

All experiments were carried out under inert atmosphere employing standard Schlenk and glovebox techniques unless otherwise stated. All chemicals were purchased from commercial sources and except for pyridine-*N*-oxide, all were used without further purification. Pyridine-*N*-oxide was recrystallized from Et_2O and sublimed at 55°C before use. All solvents were purified by a Pure Solv Solvent Purification System and stored over activated molecular sieves (3 Å). NMR spectra were recorded using a Bruker Avance III and Bruker Avance NEO 500 MHz spectrometers. ^{1}H NMR spectra were recorded at 300.13 MHz for room temperature or at 500.23 MHz for low-temperature measurements and referenced to residual protons of the NMR solvents. ^{13}C NMR spectra were obtained at 75.48 MHz for room temperature or at 125.80 MHz for low-temperature measurements and spectra were referenced to the deuterated solvent peak. $^{31}P\{^{1}H\}$ spectra were recorded at 121.49 MHz, with 85% H_3PO_4 as an external reference. The chemical shifts δ are given in ppm. The multiplicity of peaks is denoted as broad singlet (bs), singlet (s), doublet (d), triplet (t), quadruplet (q), multiplet (m), and doublet of quadruplet (dq). Coupling constants J are given in Hertz. Mass spectroscopy measurements using electron impact ionization (EI-MS) have been performed with an Agilent 5973 MSD with a push rod for direct sample measurement. IR spectra were recorded in the solid-state at a resolution of 2 cm^{-1} on a Bruker ALPHA-P Diamant ATR-FTIR. Elemental analyses (C, H, N, S) were carried out by the Department of Inorganic Chemistry at the Graz University of Technology (Heraeus Vario Elementar automatic analyzer). UV−vis spectra were recorded on a Varian Cary 50 spectrophotometer equipped with a VWR thermostat to control the temperatures of the kinetic studies using the Varian Cary WinUV software. The kinetic studies were performed at 25 °C in quartz cuvettes (d = 10 mm), and reaction solutions contained 0.1 mM of complexes, freshly prepared on the day of the experiments. Each experiment was independently performed at least three times. $[WBr_2(CO)_3(MeCN)_2]$,[1] $[MoO_2(PyS)_2]$, $[Mo_2O_3(PyS)_4]$,

$[MoO(PyS)_2(PMe_3)_2]$ and ligand salts Na(PyS) and Na(PymS) were prepared according to literature.[2] Ligand disulfides were isolated from the reaction of corresponding protonated ligands with an excess of H_2O_2, by extraction with CH_2Cl_2.

2 DFT calculations

DFT calculations were performed using the TURBOMOLE 7.4.1 software package (available from http://www.turbomole.com). Structure optimizations were carried out restraining the corresponding symmetry while utilizing the B3LYP[3–7]-D3[8] hybrid functional with a def2-TZVPPD basis set.[9,10] Thermochemical effects were calculated within the rigid rotor harmonic oscillator approximation (RRHO) at 298 K.

3 Syntheses

Synthesis of [WO_2L_2] **(1-2)**

The ligand salt (NaL) (2.05 equiv) was added portion-wise to a stirred solution of [$WBr_2(CO)_3(MeCN)_2$] (1.500 g, 2.94 mmol, 1.0 equiv) in 20 mL of CH_2Cl_2. After 45 min, the suspension was filtrated through Celite and a solution of pyridine-*N*-oxide (0.560 g, 5.88 mmol, 2.0 equiv) in 10 mL of CH_2Cl_2 was added to the filtrate. The reaction mixture was stirred for 15h with a bubbler attached. The volume of the reaction mixture was then reduced to 30 mL, 15 mL of MeCN was added and the dark yellow microcrystals were isolated upon slow solvent evaporation and cannula transfer of the excess liquid. Products were washed with cold MeCN and ether and dried in vacuo. Colorless single crystals suitable for X-ray diffraction analysis were obtained from CH_2Cl_2/MeCN mixtures at – 37°C.

[$WO_2(PyS)_2$] (1). Yield: 898 mg (70%). ^{1}H NMR (300 MHz, $CDCl_3$) δ 8.59 (ddd, J = 5.4, 1.8, 1.0 Hz, 2H, pyH-*o*), 7.67 (ddd, 8.1, 7.6, 1.8 Hz, 2H, pyH-*p*), 7.18 (ddd, J = 8.1, 1.0 Hz, 2H, pyH-*m*), 7.09 (ddd, J = 7.6, 5.4, 1.1 Hz, 2H, pyH-*m*) ppm. ^{13}C NMR (75 MHz, $CDCl_3$) δ 165.71 (s, 2C, Cq), 144.65 (s, 2C, pyC-*o*), 140.52 (s, 2C, pyC-*p*), 126.87 (s, 2C, pyC-*m*), 120.49 (s, 2C, pyC-*m*) ppm. IR (cm^{-1}): 945 (s, W=O), 902 (s,W=O). EI-MS (70 eV) *m/z*: [M]$^+$ 436.1. Anal. Calcd for $C_{10}H_8N_2O_2S_2W$ · 0.1 MeCN: C, 27.83; H, 1.90; N, 6.68; S, 14.56. Found: C, 28.15; H, 1.80; N, 6.62; S, 14.72.

[$WO_2(PymS)_2$] (2). Yield: 708 mg (55%). ^{1}H NMR (300 MHz, CD_2Cl_2): δ 8.83 – 8.89 (m, 4H, pymH-*o*), 7.21 (t, J = 5.2 Hz, 2H, pymH-*m*) ppm. ^{13}C NMR (75 MHz, CD_2Cl_2): δ 175.22 (s, 2C, Cq), 162.29 (s, 2C, pymC-*o*), 152.53 (s, 2C, pymC-*o*), 118.85 (s, 2C, pymC-*m*) ppm. IR (cm^{-1}): 953 (s, W=O), 910 (s,W=O). EI-MS (70 eV) *m/z*: [M]$^+$ 438.0. Anal. Calcd for $C_8H_6N_4O_2S_2W$ · 0.2 MeCN: C, 22.60; H, 1.49; N, 13.18; S, 14.37. Found: C, 22.84; H, 1.39; N, 12.98; S, 14.69.

Synthesis of $[WO(PMe_3)_2L_2]$ **(3-4)**

A solution of PMe_3 (280 µL, 2.75 mmol, 5 equiv) in 3 mL of CH_2Cl_2 was added to $[WO_2L_2]$ (1 equiv) dissolved in 6 mL of CH_2Cl_2. After stirring overnight, the solvent was removed and the residual powder was washed with cold MeCN and dried in vacuo. Crystals were obtained from a CH_2Cl_2/heptane mixture (**3**) or MeCN solution (**4**), both at – 37°C.

$[WO(PMe_3)_2(PyS)_2]$ (**3**) Yield: 244 mg of green microcrystals (77%). ^{1}H NMR (300 MHz, $CDCl_3$): δ 8.68 (m, 2H, pyH-*o*), 6.85 (td, J = 7.5, 1.7 Hz, 2H, pyH-*o*), 6.69 (ddd, J = 7.1, 5.7, 1.3 Hz, 2H, pyH-*m*), 6.54 (dt, J = 7.9, 1.1 Hz, 2H, pyH-*m*), 1.36 (t, $^{2}J_{HP}$ = 4.0 Hz, 18H, PCH_3) ppm. ^{13}C NMR (75 MHz, $CDCl_3$): δ 167.14 (s, 2C, Cq), 143.98 (s, 2C, pyC-o), 135.26 (s, 2C, pyC-p), 122.94 (s, 2C, pyC-m), 114.50 (s, 2C, pyC-m), 14.98 (t, $^{1}J_{CP}$ = 13.9 Hz, 6C, PCH_3) ppm. ^{31}P (121 MHz, $CDCl_3$). δ −27.66 ppm. IR (cm^{-1}): 940 (s,W=O). EI-MS (70eV) *m/z*: $[M - 2\ PMe_3]^+$ 420.0. Anal. Calcd for $C_{16}H_{26}N_2OP_2S_2W$: C, 33.58; H, 4.58; N, 4.89; S, 11.21. Found: C, 33.36; H, 4.46; N, 4.83; S, 10.82.

$[WO(PMe_3)_2(PymS)_2]$ (**4**) Yield: 199 mg of violet microcrystals (63%). ^{1}H NMR (500 MHz, $CDCl_3$, -30°C) δ 8.78 (dd, J = 5.6, 2.3 Hz, 2H, pymH-*o*), 7.87 (dd, J = 4.8, 2.3 Hz, 2H, pymH-*o*), 6.78 (t, J = 5.2 Hz, 2H, pymH-*m*), 1.37 (t, $^{2}J_{HP}$ = 4.1 Hz, 18H, PCH_3) ppm. ^{13}C NMR (126 MHz, $CDCl_3$, -30°C) δ 173.23 (s, 2C, Cq), 157.88 (s, 2C, pymC-*o*), 150.60 (s, 2C, pymC-*o*), 112.75 (s, 2C, pymC-*m*), 14.81 (t, $^{1}J_{CP}$ = 14.5 Hz, 6C, PCH_3) ppm. ^{31}P NMR (121 MHz, $CDCl_3$) δ - 27.49 ppm. IR (cm^{-1}): 939 (s,W=O). Anal. Calcd for $C_{14}H_{24}N_4OP_2S_2W$: C, 29.28; H, 4.21; N, 9.76; S, 11.17. Found: C, 29.42; H, 4.02; N, 9.86; S, 11.29.

4 Crystal Structure Determination

Crystal Structure Determination – General. All single crystal measurements were performed on a Bruker APEX-II CCD diffractometer at 100 K using Mo K_α radiation with a wavelength of 0.71073Å from an Incoatec microfocus sealed tube equipped with a multilayer monochromator. Absorption corrections were made semi-empirically from equivalents. The structures were solved by direct methods (SHELXS-97)[11] and refined by full-matrix least-squares techniques against F^2 (SHELXL-2014/6)[12]. A weighting scheme of $w = 1/[\sigma^2(F_o^2)+(aP)^2+bP]$ where $P = (F_o^2+2F_c^2)/3$ was used. The non-hydrogen atoms were refined with anisotropic displacement parameters without any constraints.

Crystal data, data collection parameters, and structure refinement details are given in Tables **S1-S2**. Further refinement information, structure and bonding parameters, SHELXL.res, and .hkl files are given in the deposited CIF file which is available free of charge from The Cambridge Crystallographic Data Centre (CCDC 2173097-2173100).

Crystallographic data

Table S1. Crystallographic data and structure refinement for complexes **1-2**.

**Crystal data**	$WO_2(PyS)_2$ (**1**)	$WO_2(PymS)_2$ (**2**)
CIF data code	MIC79	MIC146
Empirical formula	$C_{10}H_8N_2O_2S_2W$	$C_8H_6N_4O_2S_2W$
Formula weight	436.15	438.14
Crystal description	block, colourless	block, colourless
Crystal size [mm]	0.25 x 0.08 x 0.07	0.17 x 0.07 x 0.06
Temperature	100 K	100 K
Crystal system	monoclinic	monoclinic
Space group	P 2_1/c	P 2_1/c
a	6.7819(4)Å	11.5211(12)Å
b	15.1475(8)Å	12.0225(12)Å
c	11.9587(6)Å	16.4742(16)Å
α		
β	98.020(2)°	96.302(5)°
γ		
Volume	1216.49(11)$Å^3$	2268.1(4)$Å^3$
Z	4	8
Calc. Density	2.381 mg/m^3	2.566 mg/m^3
F (000)	816	1632
Linear absorption coefficient μ	9.828 mm^{-1}	10.547 mm^{-1}
Max. and min. transmission	0.746 and 0.267	0.746 and 0.487
Unit cell determination	2.69° < θ < 29.97°	2.49° < θ < 30.06°
Reflections used	9906	9812
**Data collection**		
Θ range for data collection	2.18 to 30.00°	2.10 to 25.00°
Reflections collected/ unique	31853 / 3541	75299 / 3972
Significant unique reflections	3135 with I > 2σ(I)	2659 with I > 2σ(I)
R(int), R(sigma)	0.0876, 0.0521	0.0741, 0.0802
Completeness to $θ_{max}$	99.7%	99.3%
**Refinement**		
Data/ parameters/ restraints	3541 / 156 / 0	3972 / 191 / 0
Goodness-of-fit on F^2	1.048	1.092
Final R indices [I > 2σ(I)]	R1 = 0.0264, wR2 = 0.0516	R1 = 0.0396, wR2 = 0.0747
R indices (all data)	R1 = 0.0321, wR2 = 0.0533	R1 = 0.0687, wR2 = 0.0828
Weighting scheme param. a, b	0.0095, 0.8256	0.0000, 3.4434
Largest Δ/σ in last cycle	0.001	0.001
Largest diff. peak and hole	1.527, -1.634e/$Å^3$	1.381, -1.645e/$Å^3$
**CCDC no.**	2173097	2173099

Table S2. Crystallographic data and structure refinement for complexes **3** and **4.**

**Crystal data**	$WO(PyS)_2(PMe_3)_2$ (**3**)	$WO(PymS)_2(PMe_3)_2$ (**4**)
CIF data code	MIC94B	MIC153
Empirical formula	$C_{16}H_{26}N_2OP_2S_2W$	$C_{14}H_{24}N_4OP_2S_2W$
Formula weight	572.30	574.28
Crystal description	block, green	needle, purple
Crystal size [mm]	0.21 x 0.17 x 0.16	0.27 x 0.06 x 0.04
Temperature	100 K	100 K
Crystal system	Monoclinic	Triclinic
Space group	P 2_1/n	P -1
a	10.4569(4)Å	9.0034(6)Å
b	14.1038(6)Å	12.9005(9)Å
c	14.1835(6)Å	18.0640(12)Å
α		89.696(4)°
β	90.851(2)°	82.801(3)°
γ		83.458(4)°
Volume	2091.58(15)$Å^3$	2067.9(2)$Å^3$
Z	4	4
Calc. Density	1.817 mg/m^3	1.845 mg/m^3
F (000)	1120	1120
Linear absorption coefficient μ	5.882 mm^{-1}	5.952 mm^{-1}
Max. and min. transmission	0.746 and 0.398	0.746 and 0.414
Unit cell determination	2.27° < θ < 30.10°	2.27° < θ < 30.10°
Reflections used	9439	9529
**Data collection**		
Θ range for data collection	2.04 to 30.00°	1.95 to 26.00°
Reflections collected/ unique	65153 / 6085	95707 / 8119
Significant unique reflections	4923 with I > 2σ(I)	6844 with I > 2σ(I)
R(int), R(sigma)	0.0819, 0.0464	0.0891, 0.0640
Completeness to $θ_{max}$	99.7%	99.8%
**Refinement**		
Data/ parameters/ restraints	6085 / 231 / 0	8119 / 461 / 0
Goodness-of-fit on F^2	1.036	1.044
Final R indices [I > 2σ(I)]	R1 = 0.0329, wR2 = 0.0810	R1 = 0.0412, wR2 = 0.0805
R indices (all data)	R1 = 0.0430, wR2 = 0.0886	R1 = 0.0518, wR2 = 0.0832
Weighting scheme param. a, b	0.0400, 4.1168	0.0333, 2.6538
Largest Δ/σ in last cycle	0.002	0.001
Largest diff. peak and hole	2.133, -1.891e/$Å^3$	1.604, -1.366e/$Å^3$
**CCDC no.**	2173098	2173100

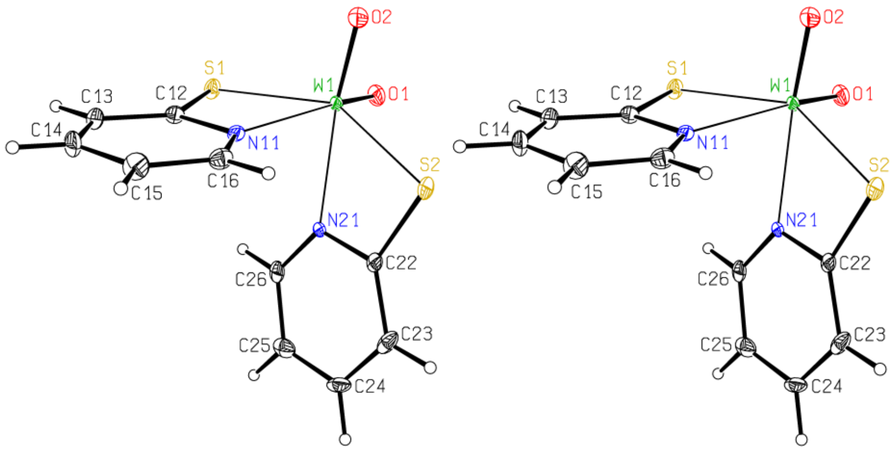

Figure S1. Stereoscopic ORTEP[13] plot of **1** showing the atomic numbering scheme. The probability ellipsoids are drawn at the 50% probability level. The H atoms are drawn with arbitrary radii.

Table S3. Selected bond lengths (Å) and angles (°) for complex **1**

W1-O1	1.721(2)	S1-W1-S2	145.19(3)
W1-O2	1.730(2)	O1-W1-O2	105.99(10)
W1-N11	2.300(2)	N11-W1-N21	76.87(8)
W1-N21	2.287(2)	C12-S1-W1	84.62(10)
W1-S1	2.4384(7)	C12-N11-C16	119.2(3)
W1-S2	2.4504(8)	C12-N11-W1	100.68(18)
S1-C12	1.774(3)	C16-N11-W1	139.9(2)
S2-C22	1.763(3)	C22-S2-W1	84.10(10)
		C22-N21-C26	120.0(3)
O1-W1-N11	157.03(9)	C22-N21-W1	100.95(18)
O2-W1-N21	156.79(10)	C26-N21-W1	139.1(2)

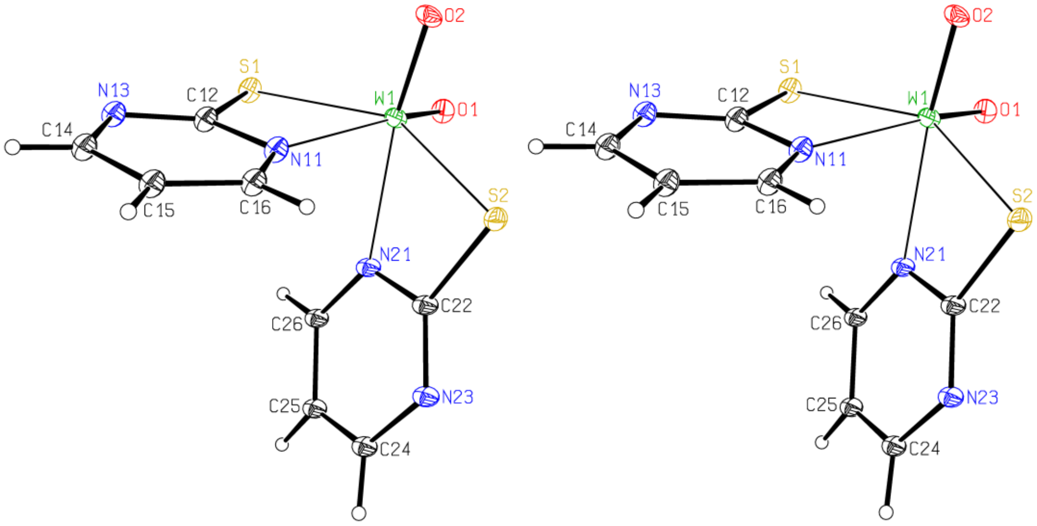

Figure S2. Stereoscopic ORTEP[13] plot of complex **A** of **2** showing the atomic numbering scheme. The probability ellipsoids are drawn at the 50% probability level. The H atoms are drawn with arbitrary radii.

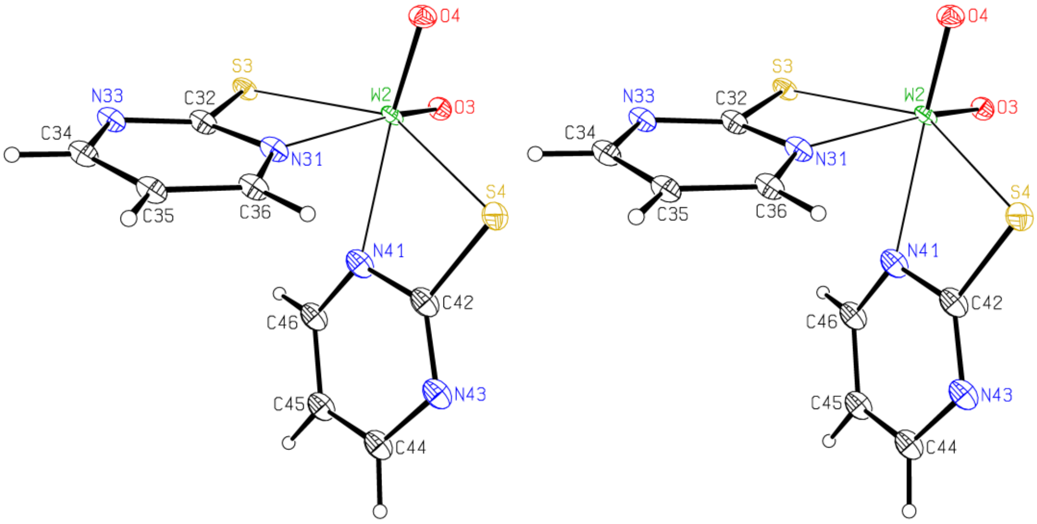

Figure S3. Stereoscopic ORTEP[13] plot of complex **B** of **2** showing the atomic numbering scheme. The probability ellipsoids are drawn at the 50% probability level. The H atoms are drawn with arbitrary radii.

Table S4. Selected bond lengths (Å) and angles (°) for complex **2**

W1-O1	1.715(7)	C12-S1-W1	84.2(4)
W1-O2	1.743(7)	C12-N11-C16	116.8(9)
W1-N11	2.308(9)	C12-N11-W1	100.6(7)
W1-N21	2.300(8)	C16-N11-W1	142.3(8)
W1-S1	2.441(3)	C22-S2-W1	84.2(4)
W1-S2	2.449(3)	C22-N21-C26	119.9(9)
S1-C12	1.784(11)	C22-N21-W1	99.0(6)
S2-C22	1.733(11)	C26-N21-W1	140.7(8)
W2-O3	1.738(7)	O3-W2-O4	106.1(3)
W2-O4	1.724(7)	O3-W2-N31	157.6(3)
W2-N31	2.319(9)	O4-W2-N41	153.1(4)
W2-N41	2.297(9)	N31-W2-N41	71.5(3)
W2-S3	2.446(3)	S3-W2-S4	145.90(9)
W2-S4	2.460(3)	C32-S3-W2	84.6(4)
S3-C32	1.734(11)	C32-N31-C36	119.5(9)
S4-C42	1.772(11)	C32-N31-W2	98.7(7)
		C36-N31-W2	141.8(8)
O1-W1-O2	104.0(3)	C42-S4-W2	83.1(4)
O1-W1-N11	157.2(3)	C42-N41-C46	117.3(10)
O2-W1-N21	156.1(4)	C42-N41-W2	99.8(7)
N11-W1-N21	74.7(3)	C46-N41-W2	141.9(9)
S1-W1-S2	145.76(9)		

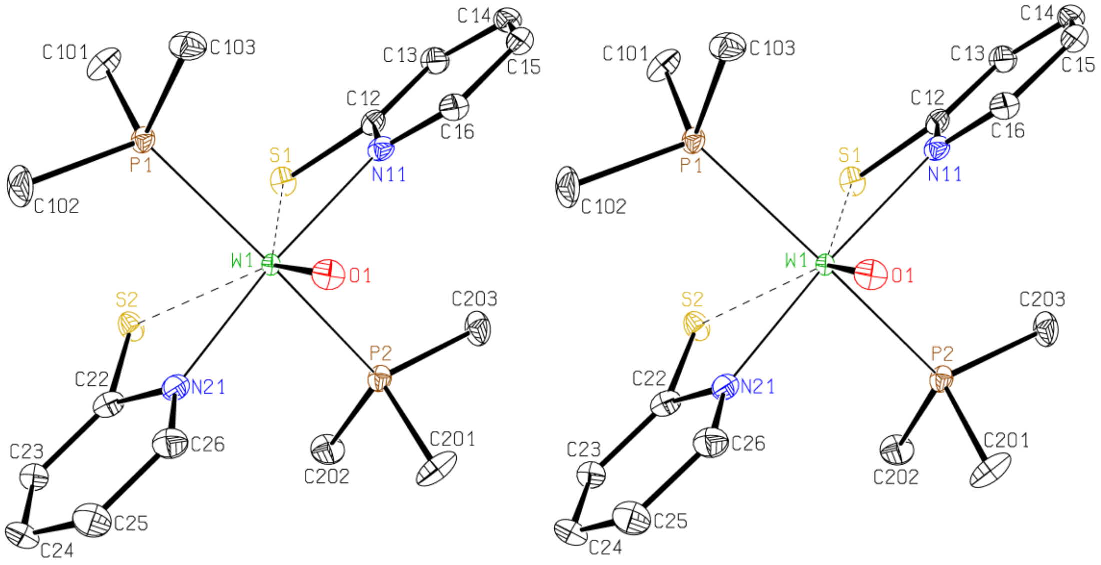

Figure S4. Stereoscopic ORTEP[13] plot of **3** showing the atomic numbering scheme. The probability ellipsoids are drawn at the 50% probability level. The rather long W–S bonds are indicated by dashed lines. The H atoms were omitted for clarity.

Table S5. Selected bond lengths [Å] and angles [°] for **3**

W1-O1	1.745(2)	N11-W1-N21	165.37(11)
W1-N11	2.205(3)	O1-W1-S1	143.75(8)
W1-N21	2.195(3)	O1-W1-S2	143.95(8)
W1-P1	2.4760(8)	N11-W1-S2	133.26(8)
W1-P2	2.4883(9)	N21-W1-S1	133.27(8)
W1-S1	2.6675(8)	C12-S1-W1	80.85(12)
W1-S2	2.6668(10)	C12-N11-C16	119.1(3)
S1-C12	1.726(4)	C12-N11-W1	108.8(2)
S2-C22	1.719(3)	C16-N11-W1	132.0(2)
		C22-S2-W1	80.74(13)
P1-W1-P2	170.08(3)	C22-N21-C26	119.4(3)
O1-W1-N11	82.57(11)	C22-N21-W1	108.9(2)
O1-W1-N21	82.80(11)	C26-N21-W1	131.5(2)
N11-W1-S1	61.32(8)		
N21-W1-S2	61.34(8)	O1-W1-P1-C101	152.75(16)
S1-W1-S2	72.31(3)	O1-W1-P2-C201	24.47(16)

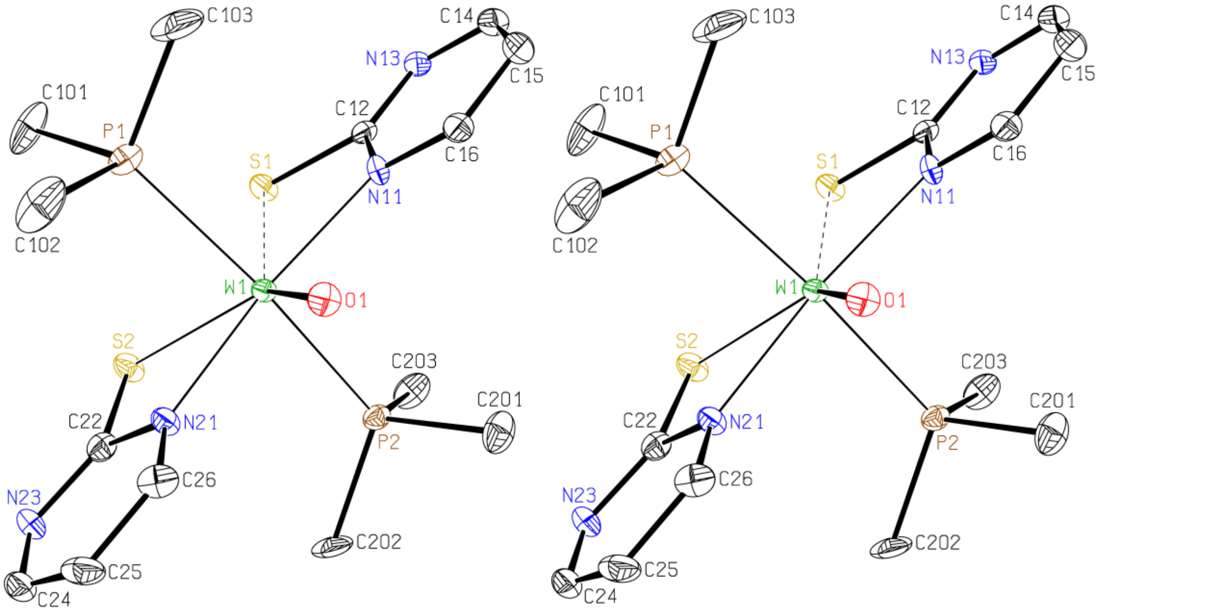

Figure S5. Stereoscopic ORTEP[13] plot of complex **A** of **4** showing the atomic numbering scheme. The probability ellipsoids are drawn at the 50% probability level. The H atoms were omitted for clarity. The rather long W–S bond is indicated by a dashed line.

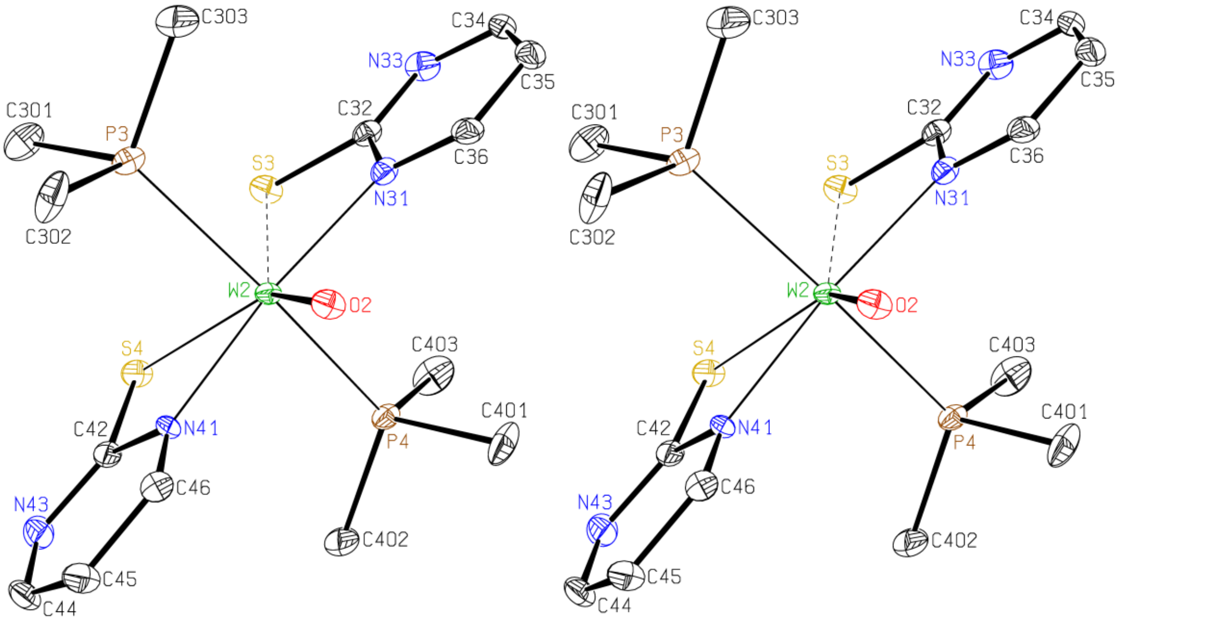

Figure S6. Stereoscopic ORTEP[13] plot of complex **B** of **4** showing the atomic numbering scheme. The probability ellipsoids are drawn at the 50% probability level. The H atoms were omitted for clarity. The rather long W–S bond is indicated by a dashed line.

Table S6. Selected bond lengths [Å] and angles [°] for **4**

W1-O1	1.727(3)
W1-N11	2.162(4)
W1-N21	2.181(4)
W1-P1	2.4909(14)
W1-P2	2.4936(14)
W1-S1	2.6825(13)
W1-S2	2.6399(12)
S1-C12	1.720(5)
S2-C22	1.710(5)
W2-O2	1.744(3)
W2-N31	2.174(4)
W2-N41	2.183(4)
W2-P3	2.4925(13)
W2-P4	2.4951(13)
W2-S3	2.6664(13)
W2-S4	2.6498(13)
S3-C32	1.714(5)
S4-C42	1.722(5)

P1-W1-P2	170.96(4)
O1-W1-N11	82.08(16)
O1-W1-N21	82.62(16)
N11-W1-S1	61.24(11)
N21-W1-S2	61.61(11)
S1-W1-S2	72.65(4)
N11-W1-N21	164.67(15)
O1-W1-S1	143.09(12)
O1-W1-S2	144.22(12)
N11-W1-S2	133.66(11)
N21-W1-S1	134.08(11)
C12-S1-W1	80.57(17)
C12-N11-C16	116.7(4)
C12-N11-W1	110.4(3)
C16-N11-W1	132.9(4)
C22-S2-W1	81.26(17)
C22-N21-C26	118.5(4)
C22-N21-W1	108.7(3)
C26-N21-W1	132.7(3)
P3-W2-P4	171.03(4)
O2-W2-N31	83.01(16)
O2-W2-N41	82.82(16)
N31-W2-S3	61.48(11)
N41-W2-S4	61.49(11)
S3-W2-S4	71.60(4)
N31-W2-N41	165.76(15)
O2-W2-S3	144.18(12)
O2-W2-S4	144.22(12)
N31-W2-S4	132.56(11)
N41-W2-S3	132.76(11)
C32-S3-W2	80.46(18)
C32-N31-C36	117.5(5)
C32-N31-W2	109.2(3)
C36-N31-W2	133.3(4)
C42-S4-W2	81.06(17)
C42-N41-C46	117.9(4)
C42-N41-W2	109.3(3)
C46-N41-W2	132.8(3)
O1-W1-P1-C101	-172.9(2)
O1-W1-P2-C201	-21.2(2)
O2-W2-P3-C301	-165.3(2)
O2-W2-P4-C401	-15.1(2)

5 NMR spectra

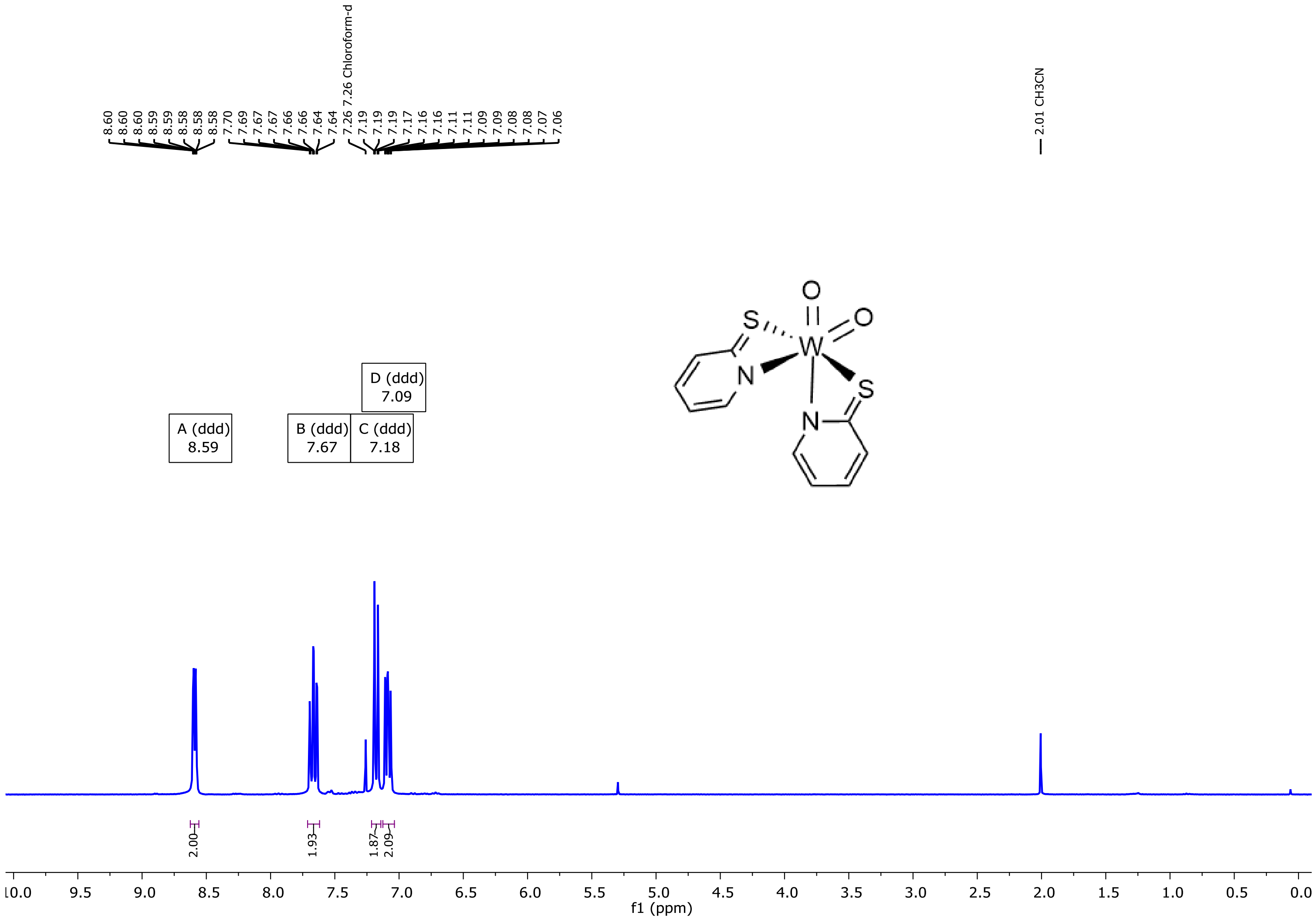

Figure S7. ^{1}H NMR spectrum of $[WO_2(PyS)_2]$ (**1**) in $CDCl_3$.

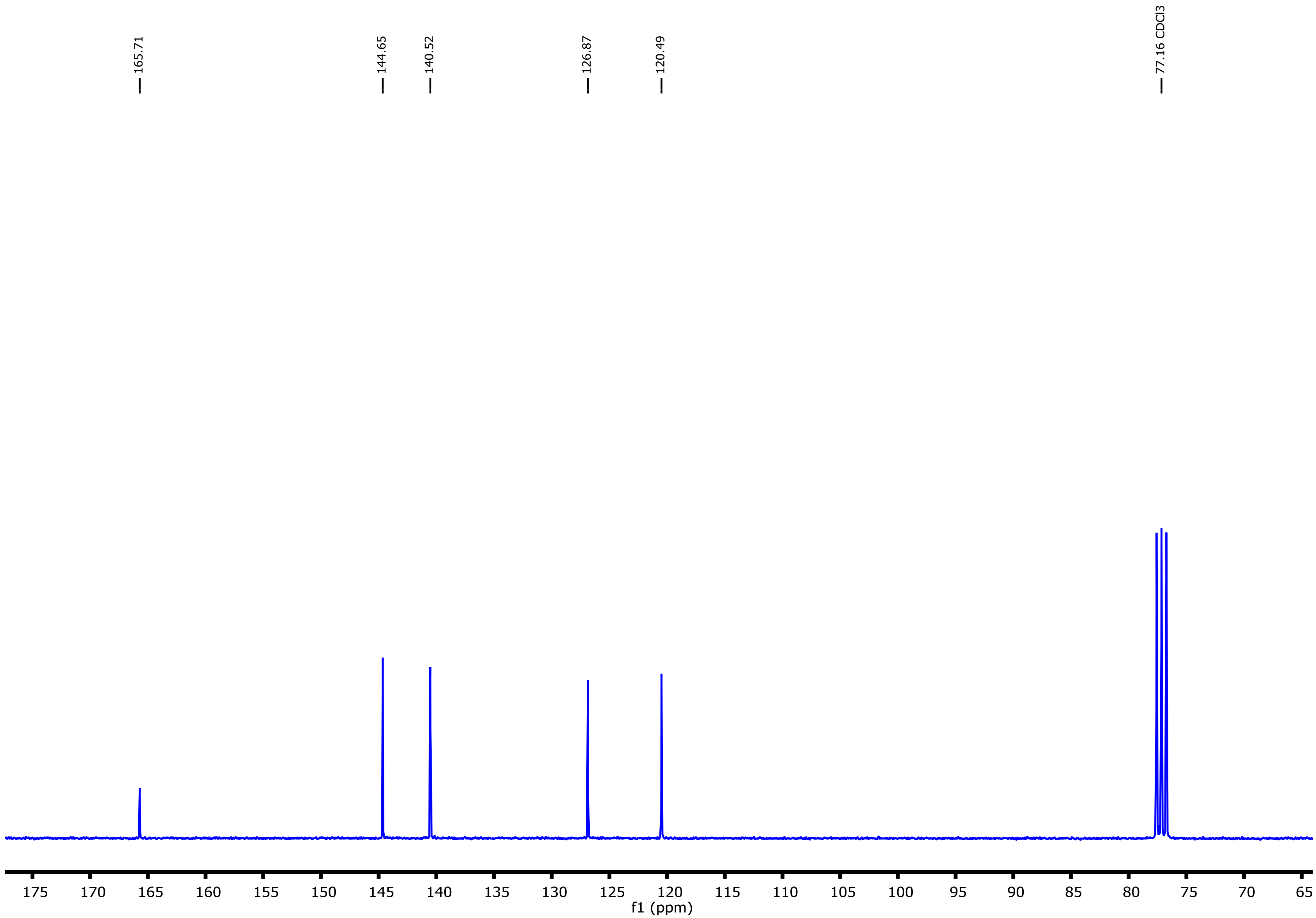

Figure S8. ^{13}C NMR spectrum of $[WO_2(PyS)_2]$ (**1**) in $CDCl_3$.

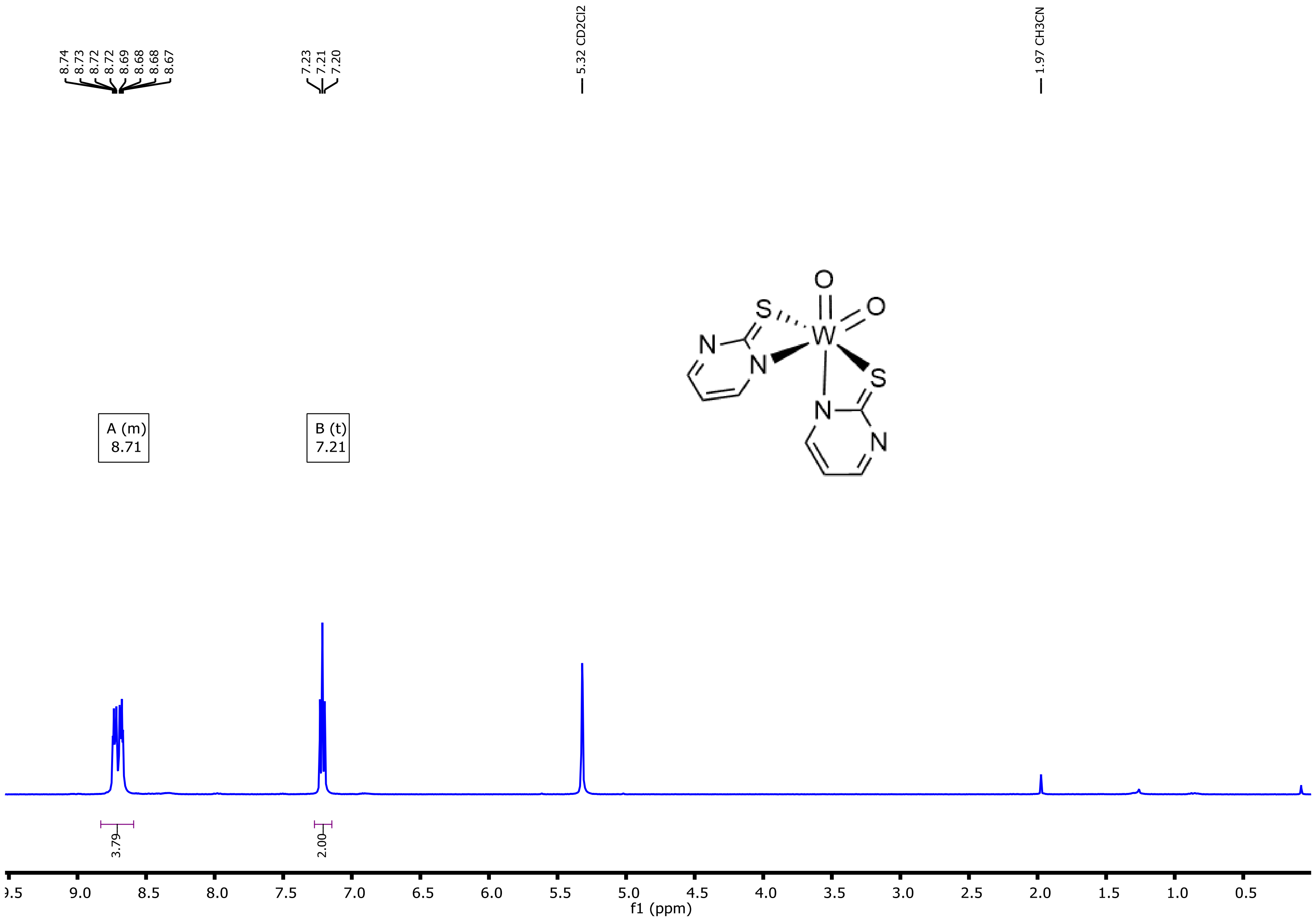

Figure S9. ^{1}H NMR spectrum of $[WO_2(PymS)_2]$ (**2**) in CD_2Cl_2.

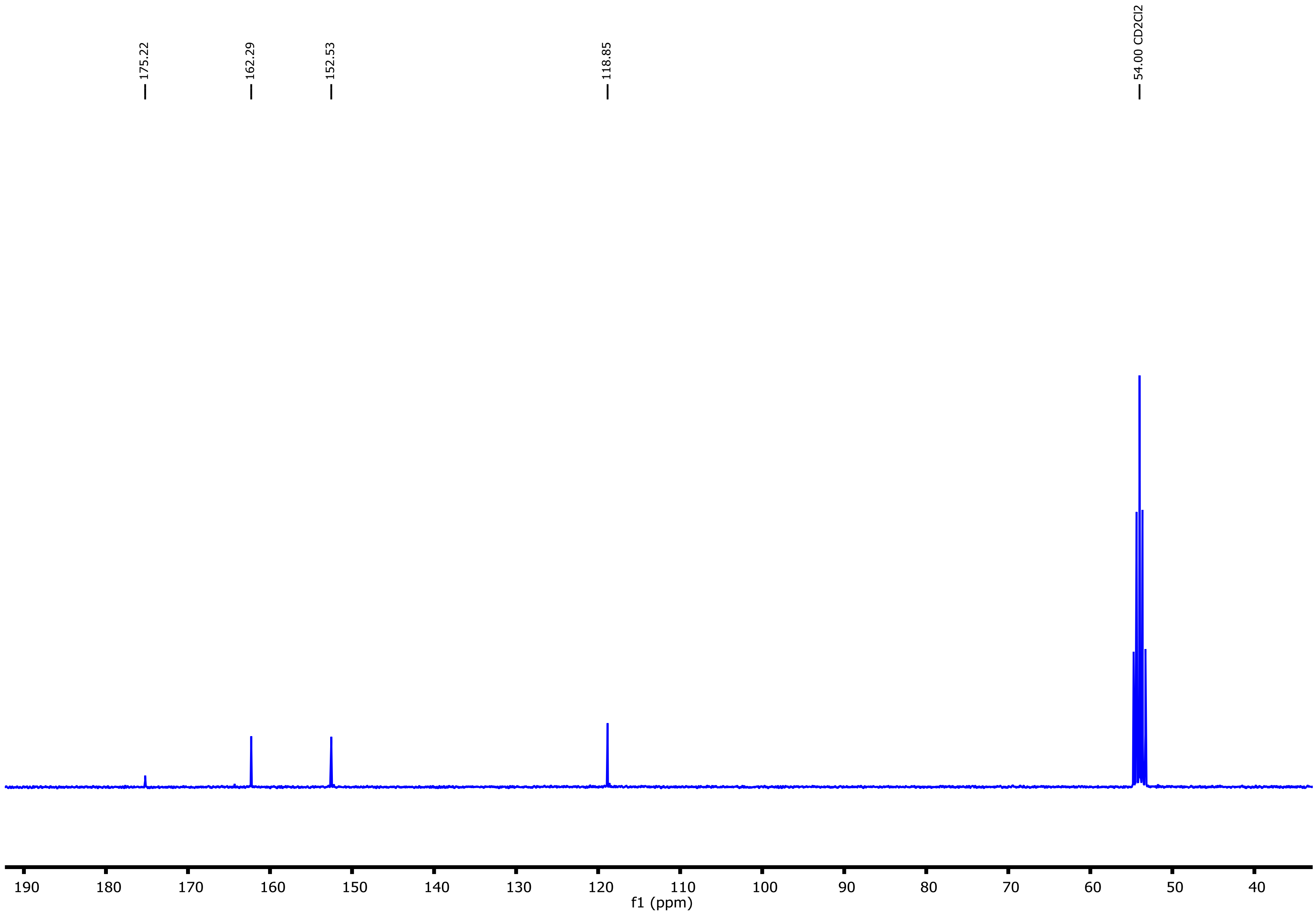

Figure S10. ^{13}C NMR spectrum of $[WO_2(PymS)_2]$ (**2**) in CD_2Cl_2.

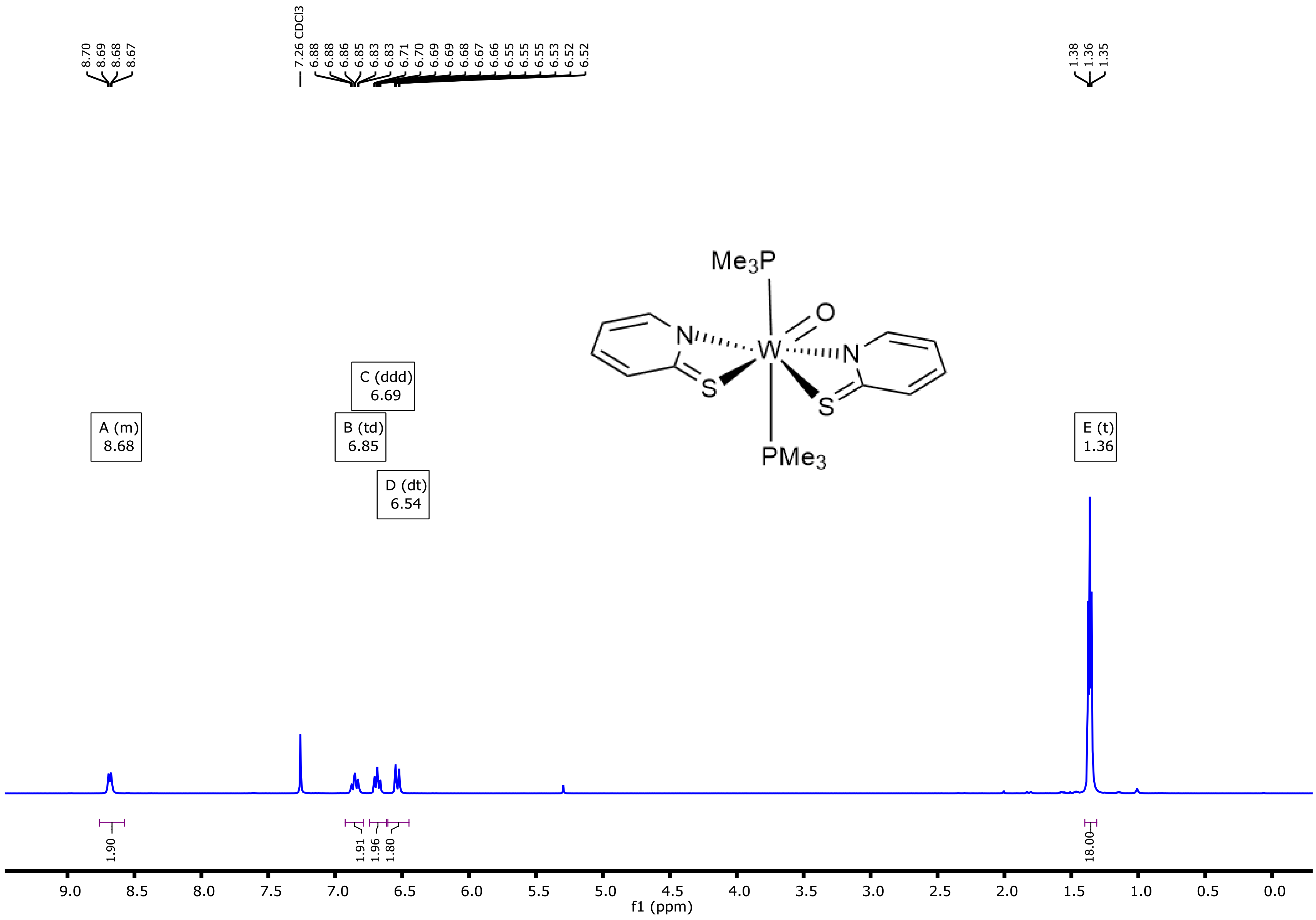

Figure S11. ^{1}H NMR spectrum of $[WO(PMe_3)_2(PyS)_2]$ (**3**) in $CDCl_3$.

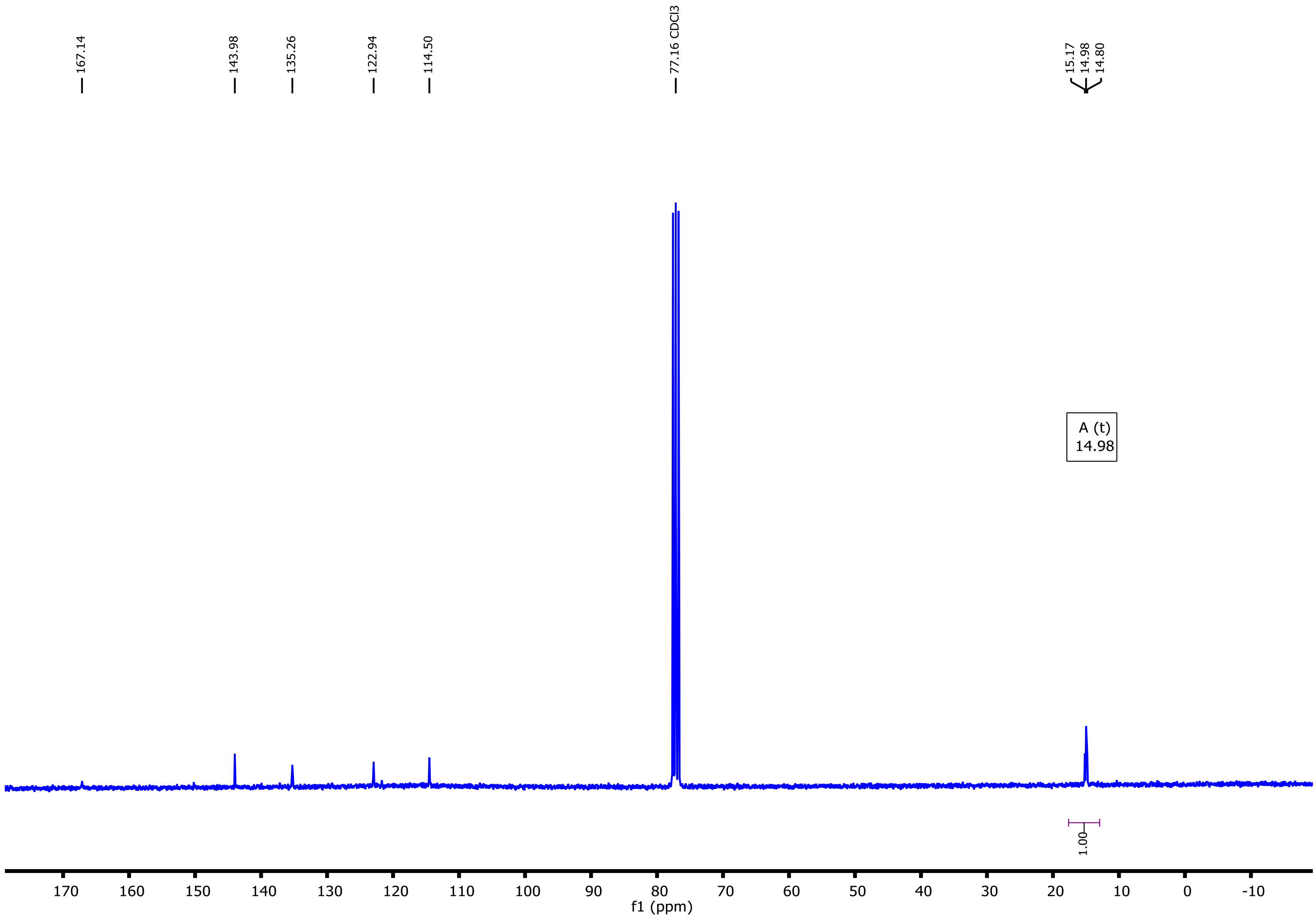

Figure S12. ^{13}C NMR spectrum of $[WO(PMe_3)_2(PyS)_2]$ (**3**) in $CDCl_3$.

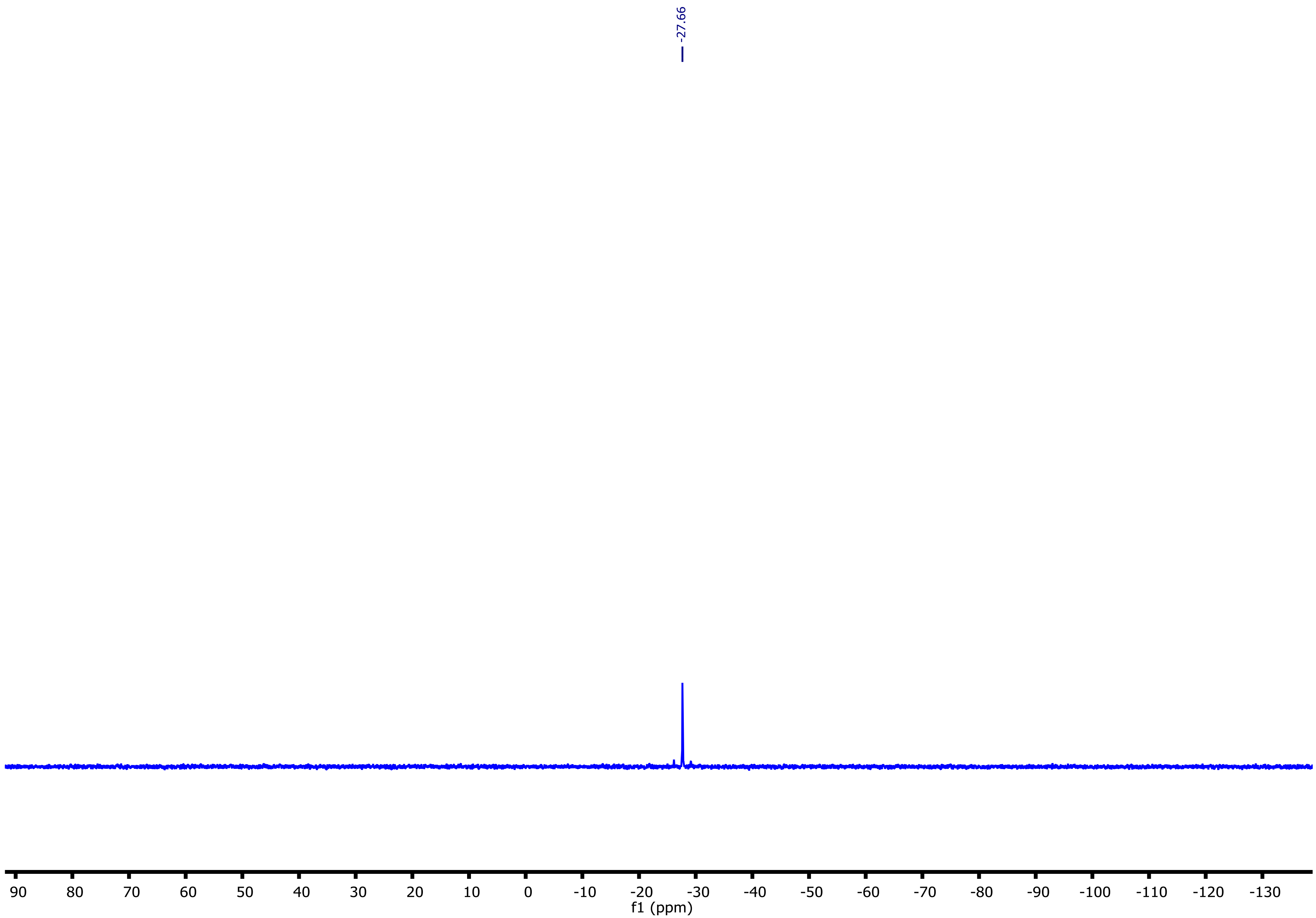

Figure S13. ^{31}P NMR spectrum of [WO(PMe_3)$_2$(PyS)$_2$] (**3**) in $CDCl_3$.

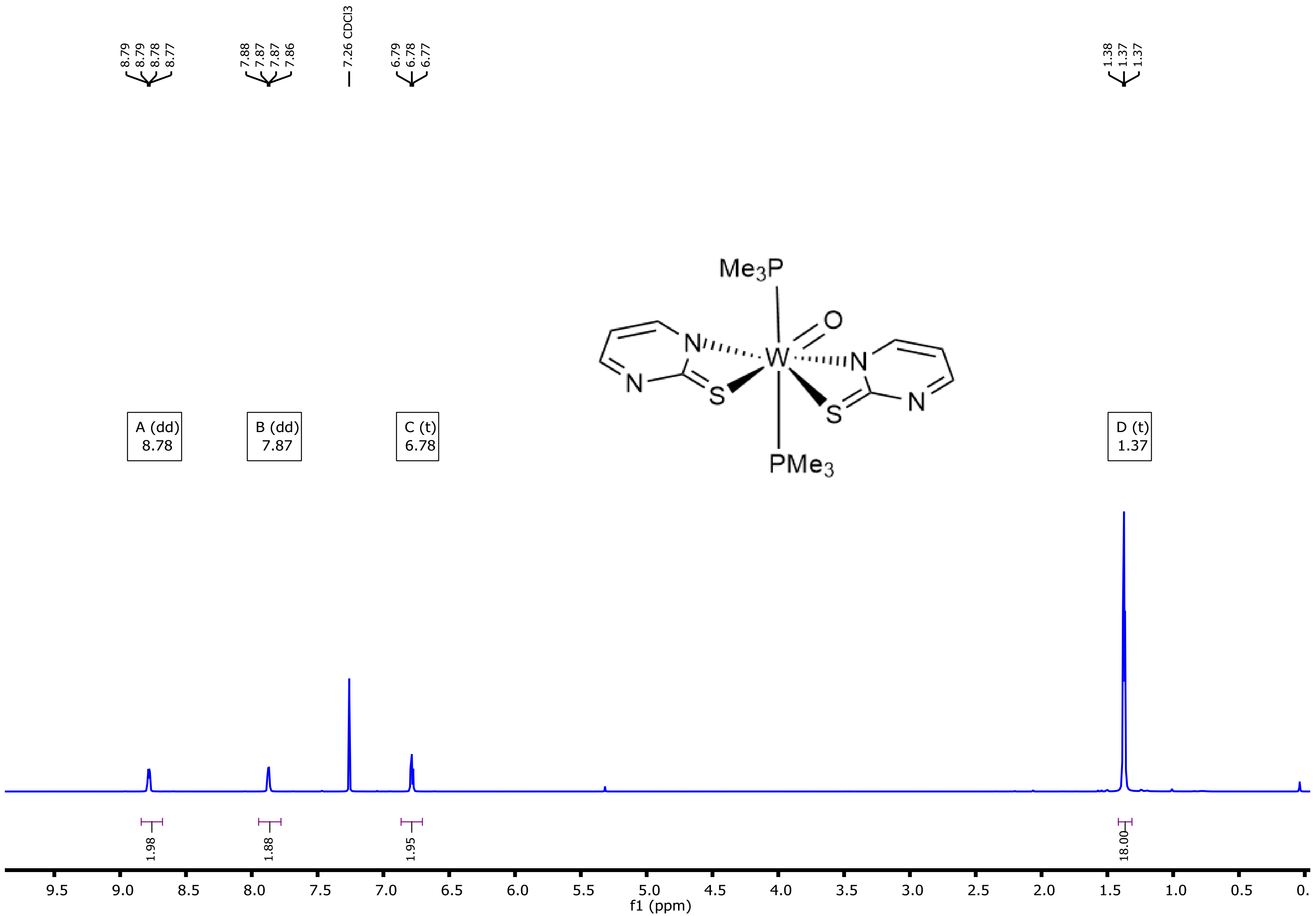

Figure S14. ^{1}H NMR spectrum of $[WO(PMe_3)_2(PymS)_2]$ (**4**) in $CDCl_3$ at -30°C.

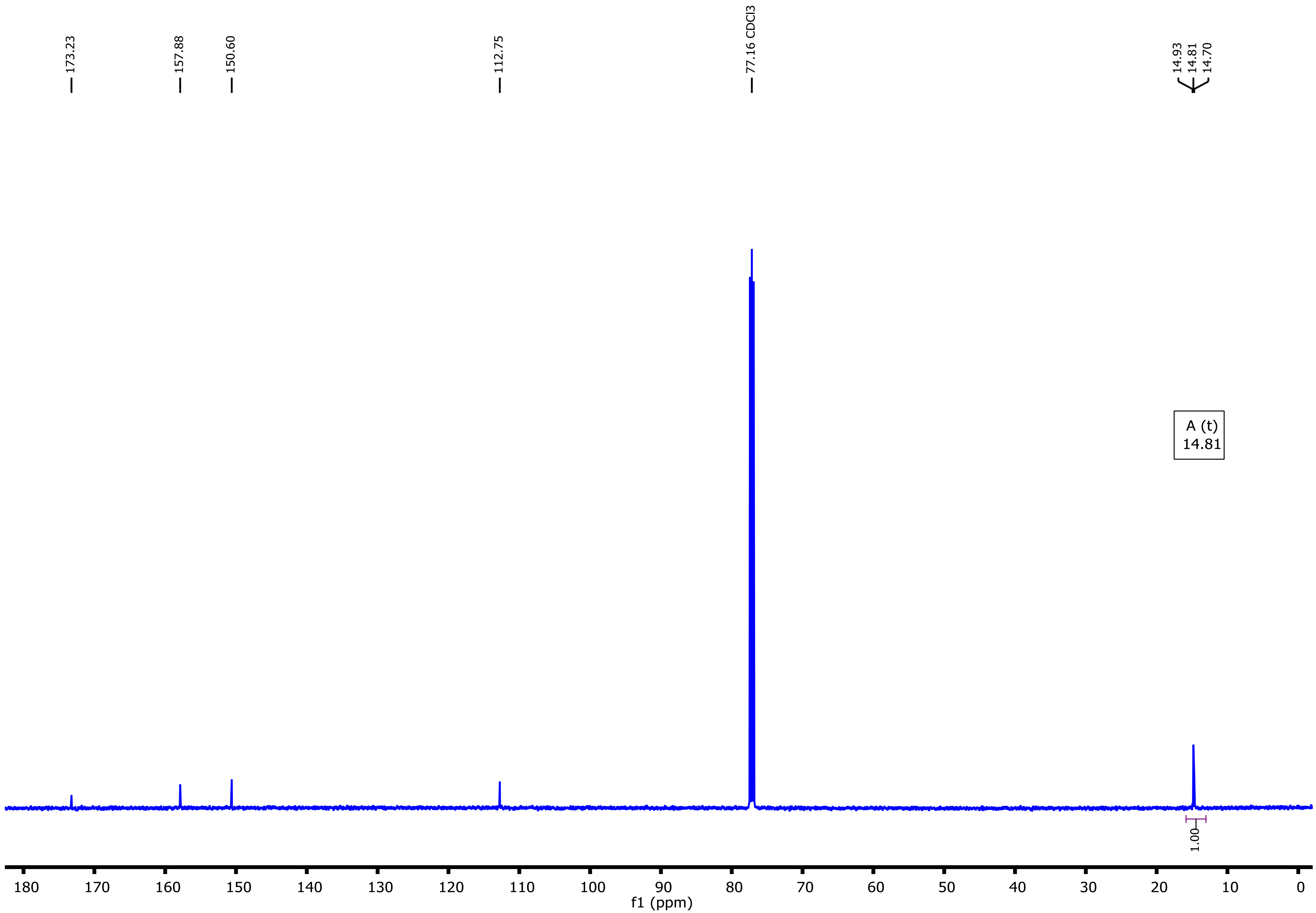

Figure S15. ^{13}C NMR spectrum of $[WO(PMe_3)_2(PymS)_2]$ (**4**) in $CDCl_3$ at -30°C.

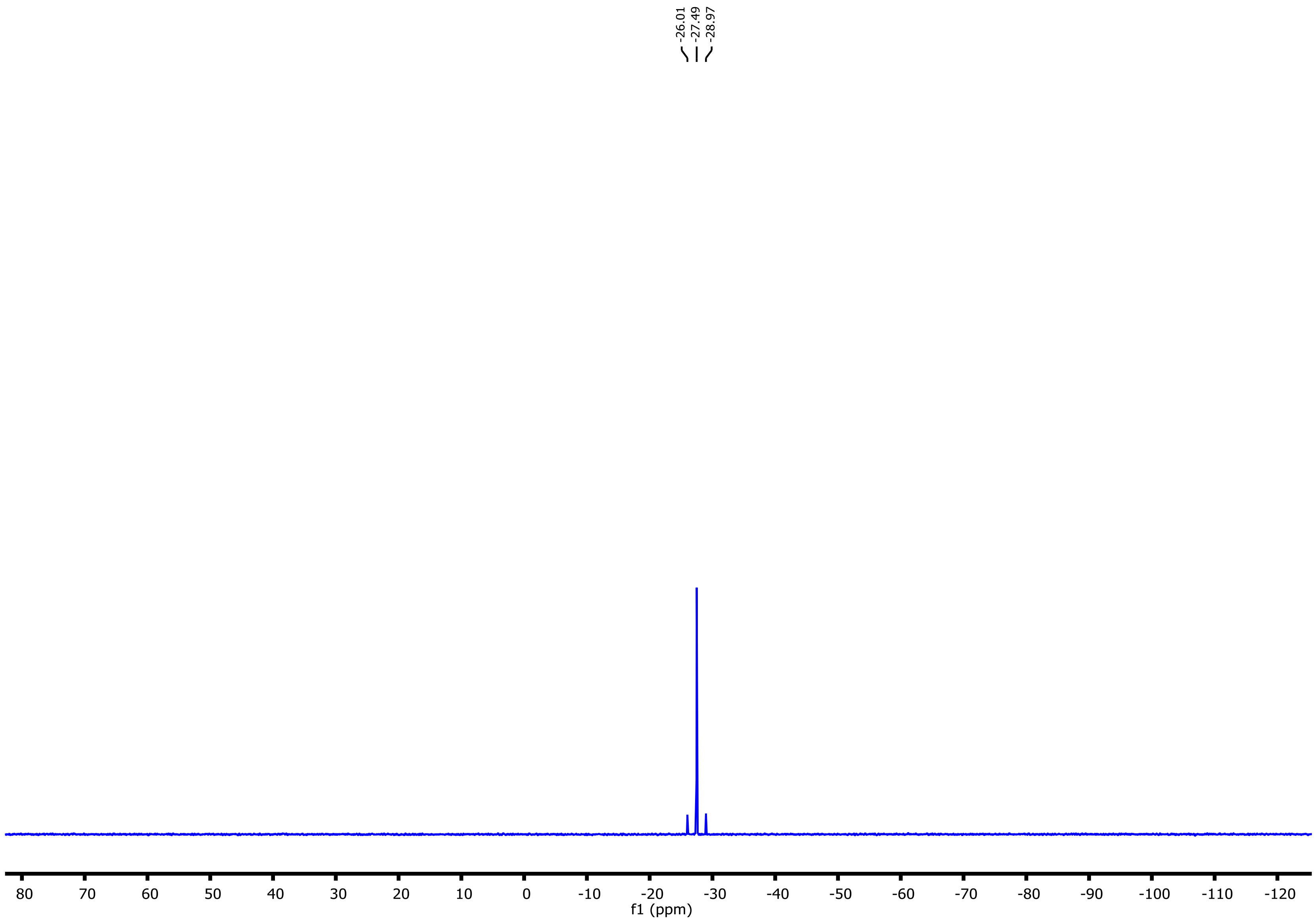

Figure S16. ^{31}P NMR spectrum of $[WO(PMe_3)_2(PymS)_2]$ (**4**) in $CDCl_3$ at -30°C.

6 UV-VIS data

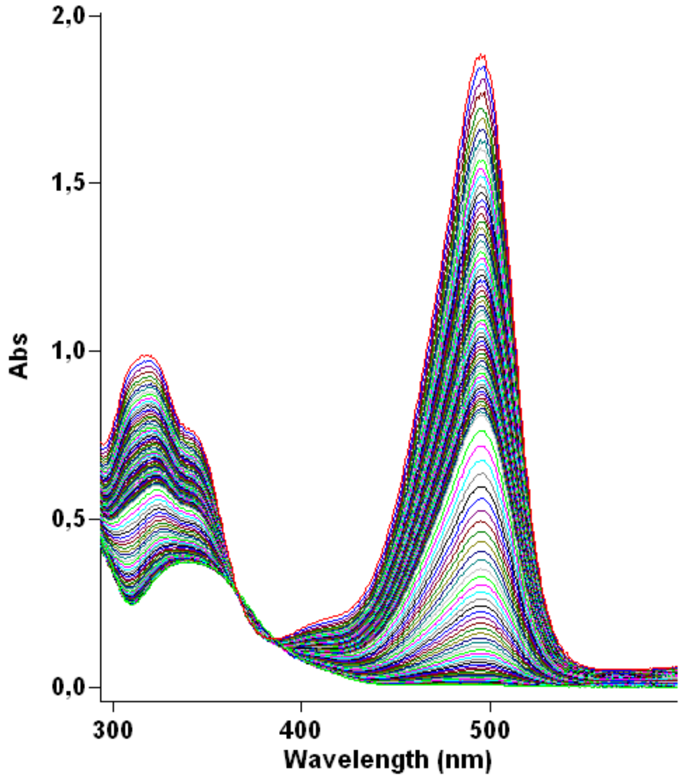

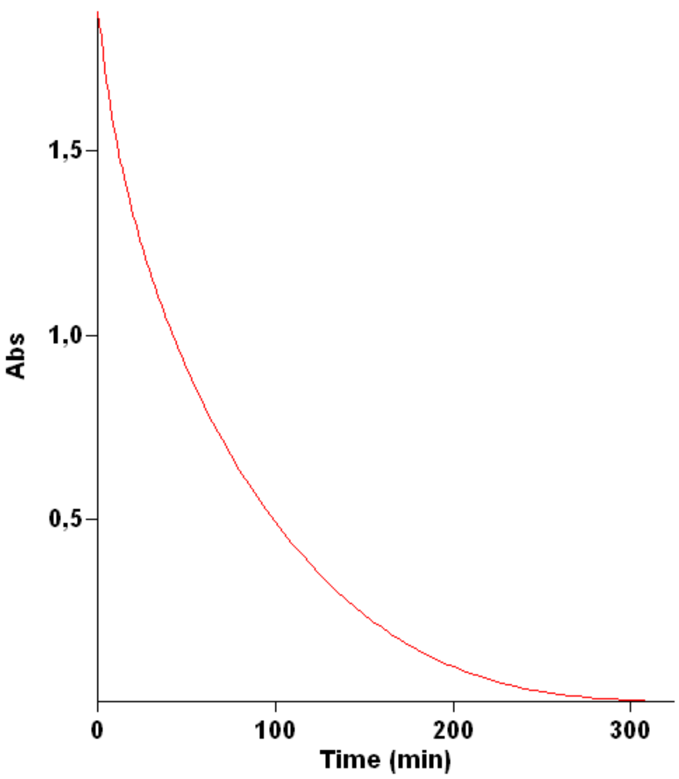

Figure S17. Left: [WO(PyS)$_2$(PMe$_3$)$_2$] (**4**) (λ^1_{max} = 320 nm; λ^2_{max} = 495 nm) reacts to [WO$_2$(PyS)$_2$] (λ_{max} = 335 nm) during 5 h; Right: Decrease of absorbance intensity at 495 nm over 5h

7 References

(1) Vidovič, C.; Peschel, L. M.; Buchsteiner, M.; Belaj, F.; Mösch-Zanetti, N. C. Structural Mimics of Acetylene Hydratase: Tungsten Complexes Capable of Intramolecular Nucleophilic Attack on Acetylene. *Chem. Eur. J.* **2019**, *25* (63), 14267–14272.

(2) Ehweiner, M. A.; Wiedemaier, F.; Belaj, F.; Mösch-Zanetti, N. C. Oxygen Atom Transfer Reactivity of Molybdenum(VI) Complexes Employing Pyrimidine- and Pyridine-2-thiolate Ligands. *Inorg. Chem.* **2020**, *59* (19), 14577–14593.

(3) Vosko, S. H.; Wilk, L.; Nusair, M. Accurate spin-dependent electron liquid correlation energies for local spin density calculations: a critical analysis. *Can. J. Phys.* **1980**, *58* (8), 1200–1211.

(4) Lee, C.; Yang, W.; Parr, R. G. Development of the Colle-Salvetti correlation-energy formula into a functional of the electron density. *Phys. Rev. B* **1988**, *37* (2), 785–789.

(5) Becke, A. D. Density-functional exchange-energy approximation with correct asymptotic behavior. *PRA, General physics* **1988**, *38* (6), 3098–3100.

(6) Becke, A. D. Density-functional thermochemistry. III. The role of exact exchange. *J. Chem. Phys.* **1993**, *98* (7), 5648–5652.

(7) Stephens, P. J.; Devlin, F. J.; Chabalowski, C. F.; Frisch, M. J. Ab Initio Calculation of Vibrational Absorption and Circular Dichroism Spectra Using Density Functional Force Fields. *J. Phys. Chem.* **1994**, *98* (45), 11623–11627.

(8) Grimme, S.; Anthony, J.; Ehrlich, S.; Krieg, H. A consistent and accurate ab initio parametrization of density functional dispersion correction (DFT-D) for the 94 elements H-Pu. *J. Chem. Phys.* **2010**, *132* (15), 154104.

(9) Weigend, F.; Furche, F.; Ahlrichs, R. Gaussian basis sets of quadruple zeta valence quality for atoms H–Kr. *J. Chem. Phys.* **2003**, *119* (24), 12753–12762.

(10) Rappoport, D.; Furche, F. Property-optimized gaussian basis sets for molecular response calculations. *J. Chem. Phys.* **2010**, *133* (13), 134105.

(11) Sheldrick, G. M. A short history of SHELX. *Acta Cryst.* **2008**, *A64*, 112–122.

(12) Sheldrick, G. M. Crystal structure refinement with SHELXL. *Acta Cryst.* **2015**, *71*, 3–8.

(13) Johnson, C. K. *ORTEP. Report ORNL-3794*.
